# The Role of Zebrafish and Laboratory Rodents in Schizophrenia Research

**DOI:** 10.3389/fpsyt.2020.00703

**Published:** 2020-08-27

**Authors:** Veronika Langova, Karel Vales, Petra Horka, Jiri Horacek

**Affiliations:** ^1^Translational Neuroscience, National Institute of Mental Health, Prague, Czechia; ^2^Third Faculty of Medicine, Charles University, Prague, Czechia; ^3^Institute for Environmental Studies, Faculty of Science, Charles University, Prague, Czechia; ^4^Brain Electrophysiology, National Institute of Mental Health, Prague, Czechia

**Keywords:** schizophrenia, animal models, zebrafish, laboratory rodents, model validity, schizophrenia symptoms, neurobiology

## Abstract

Schizophrenia is a severe disorder characterized by positive, negative and cognitive symptoms, which are still not fully understood. The development of efficient antipsychotics requires animal models of a strong validity, therefore the aims of the article were to summarize the construct, face and predictive validity of schizophrenia models based on rodents and zebrafish, to compare the advantages and disadvantages of these models, and to propose future directions in schizophrenia modeling and indicate when it is reasonable to combine these models. The advantages of rodent models stem primarily from the high homology between rodent and human physiology, neurochemistry, brain morphology and circuitry. The advantages of zebrafish models stem in the high fecundity, fast development and transparency of the embryo. Disadvantages of both models originate in behavioral repertoires not allowing specific symptoms to be modeled, even when the models are combined. Especially modeling the verbal component of certain positive, negative and cognitive symptoms is currently impossible.

## Introduction

Schizophrenia is a severe mental disorder affecting approximately 1% of the worldwide adult population and often ending fatally (1–3). The pathophysiology stems from an imbalance in excitatory and inhibitory neurotransmission with consequent dysregulation of neuromodulation resulting in a set of positive, negative and cognitive symptoms (4). Positive symptoms include hallucinations, delusions and disorganized speech. Negative symptoms originate from emotional flattening related to abulia, alogia and anhedonia, leading to social withdrawal. Cognitive deficits consist of disrupted sensorimotor gating, learning and executive functions (5). The exact neurobiological mechanisms underlying schizophrenia symptoms are still not known and the knowledge gaps substantially limit the development of effective treatment, especially the cure of negative and cognitive symptoms (6). Animal models of a high construct validity evaluating the ability of the model to reconstruct the etiopathogenesis of the disease, a high face validity reflecting the similarity of modeled symptoms with the disorder expression, and a high predictive validity evaluating the possibility to normalize elicited symptoms by antipsychotic drugs, are required (7, 8).

A plethora of animal models using pharmacological, neurodevelopmental and other strategies has been created. The most commonly used models are based on laboratory rodents, although fish, mostly zebrafish (*Danio rerio*, *D. rerio*) species (8), have recently become popular. Rodents and zebrafish possess undisputed qualities, although their use in schizophrenia modeling often does not allow a clear interpretation of results in behavioral and other analyses. The models can be often combined to bridge at least some of these gaps, though some crucial questions remain unanswered. Being mammals, rats and mice display similarities to human physiology, brain morphology and behavior. However, the lack of speech (or its equivalent) substantially limits modeling the positive symptoms only to non-verbal markers such as hyperlocomotion or stereotypy, and negative symptoms such as thigmotaxis (keeping contact with objects such as walls), decreased exploratory behavior or reduced social interaction, when modeling some specific symptoms is completely restricted (9). The zebrafish model represents a highly significant tool in pharmacological and toxicological as well as behavioral research (10). The limitations of using zebrafish arise from differences between human’s and fish’ physiology, which are more pronounced that between human’s and rodent’s, and as well as in rodents, from a lack of possibility to directly observe communicational disabilities. However, zebrafish is a social species and even though exact mechanisms and motivations of the social behavior are not fully explored, several tasks investigating the social behavior have been developed, offering utilization of zebrafish in neuropsychopharmacological research. These include social preference test (SPT), used as well in rodent’s experiments, mirror biting test (MBT) or analyses of a group behavior known as shoaling (11–15).

The aims of the present article are as follows. Firstly, to summarize the construct, face and predictive validity of schizophrenia models based on rodents and zebrafish. To this end, we also discuss recent advances in modeling techniques. Secondly, to compare the advantages and disadvantages of these models, and thirdly, to propose future directions in schizophrenia modeling and determine when it is reasonable to combine both models.

## Construct Validity of Rodent and Zebrafish Models: Genetic and Environmental Causes of Schizophrenia and Their Neurodevelopmental and Neurochemical Consequences

Schizophrenia originates in a combination of neurodevelopmental and neurodegenerative factors and is often genetically predetermined. Despite the early cause, schizophrenia usually does not appear until the adulthood (4, 7, 16). Distinct risk factors converge at a disruption in neuromodulation and the balance of excitatory and inhibitory neurotransmission, when an especially vulnerable target is the corticostriatal and corticolimbic circuitry including the prefrontal, cingulate and temporal cortex, hippocampus, amygdala, basal ganglia, thalamus, as well as the ventral tegmental area (VTA), and are expressed in altered emotional reaction seen in schizophrenia or bipolar disorder (17–21).

### Modeling Genetic Factors

Given the very high heritability index (more than 70%) it is not surprising that more than a hundred altered genetic loci have been identified to be associated with psychosis and the number of revealed genetic factors still increases (22, 23). These include genes of proteins affecting neurotransmission (e.g. neurotransmitter and neuromodulator receptors, t-SNARE domain containing 1); development, maturation and migration of neurons and glial cells (disrupted-in-schizophrenia 1 (disc1), neuregulin 1); synaptic development, plasticity and transport (voltage-sensitive chloride channel 3, neuregulin 1); or immunity (the major histocompatibility complex region on chromosome 6) (24–27).

#### Techniques of Genetic Engineering in Zebrafish and Rodents

The increasing popularity in the use of zebrafish in behavioral genetics is mostly based on their high breeding potential, suited for large-scale forward as well as reverse genetic studies (including genetic screens), the transparency of an embryo, allowing a direct observation of development, and a short life cycle. These and more zebrafish traits alone and in combination among numerous technical advantages offer also an opportunity to investigate some complex schizophrenia-related issues including identifying individual mutations and their relation to developmental disturbations and in general to schizophrenia markers, impact of genetic and environmental factors related to schizophrenia on offsprings and next generations, including the heritability of epigenetic abnormalities; differences between healthy and schizophrenia-like individuals throughout the development and aging or relation of early-life imbalance in neurochemistry (elicited by either genetic or environmental factors) and schizophrenia-like symptoms in adulthood.

From the technical aspect, zebrafish numerous populations allow effective investigation of genome, transcriptome or proteome and its manipulations. Regularly spawning up to two hundred viable eggs per week all year round makes zebrafish one of the most popular model organisms in genetics in general (11, 28, 29). In comparison, laboratory mice give birth to 2–8 litters containing on average 6–8 pups (30) and laboratory rats to 7–9 litters, each containing 6–12 pups depending on the strain. Identifying genotype can be performed by various sequencing techniques including next generation sequencing, such as RNA-sequencing (RNA-seq) (31). Example of a technique combining recombination and genomic analysis is targeting induced local lesions in genomes (TILLING) (11, 28, 29, 32, 33). Besides that, targeted mutations in zebrafish often generated by Gal4-upstream activating sequence (UAS) system (34). In both, zebrafish and rodent models, can be targeted mutations obtained by Cre/lox recombination (35–37), clustered regularly interspaced short palindromic repeats (CRISPR) and associated systems, zinc finger nucleases (ZFNs) and transcription activator-like effector nucleases (TALENs) (38, 39). To analyse contribution of individual genes or combination of two or more genes to a particular phenotype can be compared heterozygous and dominant and recessive homozygous animals. Moreover, studying combination of several mutations not only gives information about a particular disease, but in final also contributes to the general knowledge about the cooperation between the individual genes. Examples of genetic models related to schizophrenia include mouse models lacking genes encoding proteins involved in neurotransmitter’s metabolism (catechol-O-methyl transferase) and proteins involved in synaptic transport and plasticity (dysbindin-1, activity-regulated cytoskeleton-associated protein), which also provides an insight to participation of individual proteins in neurotransmission and mutual influence on their expression or may serve to reveal common traits shared with at the first sight different diseases, such as Parkinson’s disease (40–46).

Complementary to rodent models were developed zebrafish genetic models of schizophrenia, including models studying contribution of genes encoding proteins involved in metabolism and function of individual neurotransmitters (tyrosine hydroxylase, D_4_ receptor) or proteins controlling development (Disc1) (47–51).

Regulation of gene expression at the translational level is in both model organisms performed by micro-RNA molecules, currently popular are morpholino antisense oligonucleotides (MO). Example of using MO in relation to schizophrenia is the interaction of MO with microRNA molecules regulating neurodevelopment and neurotransmission (52–55).

Despite the fact, that most of the recombinant and subsequent visualisation techniques are available and suitable for both, zebrafish and rodent, models, zebrafish genetic models are more easily accessible. The external development of zebrafish allows genetic modifications to be easily performed on fertilized eggs or the early stage of embryos (34) and the non-invasive observation of the neurodevelopment and neuronal firing in real time using variety of brain imaging techniques including Ca^2+^ imaging or visualization of fluorescent proteins (56, 57). In comparison, generation of recombinant rodents is much more complicated and requires surgery including anesthesia and manipulation with the mother and a transplantation of genetically modified embryonic stem cells into the early stage of the embryo or by direct transfer of the gene into a specific embryonic tissue (37, 58).

On the other hand, the external development of zebrafish may limit studying the interaction between genetic and certain environmental effects on the development of a psychiatric illness. Such environmental effects impacting prenatal development in mammals arise from changes in maternal physiology during a pregnancy, and therefore can’t be modeled in species developing externally, especially when lacking a parental care. Examples of these parameters are changes in level of maternal glucocorticoids or quality of the nutrition during a pregnancy. On the other hand, similarities between rodent and human physiology, including the pregnancy and parental care, allow to overcome these obstacles. Moreover, rodent models are still irreplaceable in modeling effects of genetics on brain morphology or more complex behavior, such as social withdrawal, and the underlying neurobiological substrate, often highly similar to humans. For a better insight into the relationship between human’s genetics and health, a primate genetic model based on the mouse lemur (*Microcebus murinus*) has been currently investigated (59).

### Modeling Environmental Factors

Compared to the above mentioned high heritability, the contribution of environmental factors up to 30% is relatively low, though still significant (60). However, the impact of environmental factors can be higher, especially in risk environments, and enhanced or reduced by interactions with particular genetic factors (3, 61–63).

Humans are especially sensitive to environmental factors contributing to schizophrenia during the late second and the third trimester of prenatal development, which represent a period of massive neurogenesis and creation of neural connections and circuits or during. Other developmental windows occur during a puberty and early adolescence (corresponding to the typical onset of schizophrenia) (7, 64–67), when the central nervous system matures on a morphological as well as functional level, including strengthening of the circuitry by the formation of new connections and myelination of individual cells, but also axonal and synaptic pruning (18, 68, 69).

#### Techniques of Modeling Environmental Factors in Zebrafish and Rodents

Modeling environmental factors of psychiatric disorders is based on ethological observations of the species in its natural habitat determining e.g. the character of the stressor or timing of foraging, as well as performance and physiological features of the species, determining e.g. the timing of humoral treatment, immune challenge or changes in diet composition.

##### Stress Modeled in Mammals and Zebrafish

Excessive stress reactivity is a common trait for many mental illnesses including schizophrenia (70). One of possible theories explaining mechanisms of increased vulnerability to stress and other common traits of mental disorders offers the early life stress theory, assuming that altered activity of the hypothalamus–pituitary–adrenal (HPA) axis in early development results in malformations or at least mental alterations, some of which are related to schizophrenia (71). The stress reaction is an evolutionary conservative mechanism and despite some differences highly homological between mammals and teleost fish. The analogy to mammalian adrenals in zebrafish is the head kidney (72).

The main glucocorticoid in humans and zebrafish is cortisol, and in the case of rats and mice corticosterone. The homological pathway to mammalian HPA axis in zebrafish is the hypothalamus–pituitary–interrenal (HPI) axis (73).

In mammals, the placenta divides the foetal and maternal environment and contains 11β-hydroxylase 2 (HSD11B2), which defends the foetus against excessive levels of maternal glucocorticoids. However, in case of very severe acute stress or chronic stress, excessive amounts of glucocorticoids are not effectively deactivated and affect overall foetal development, including neurodevelopment.

Commonly used paradigms testing prenatal stress are repeated restraint of a pregnant mother in a tight container as predictable stress or unpredictable variable stress including several distinctive and successive stress paradigms (8). Alternatively, animals can be exposed to synthetic glucocorticoid dexamethasone under laboratory conditions; however, the effect on an adult schizophrenia-like phenotype is less expressed compared to exposure to excessive corticosterone release during actual behavioral stress (74). Postnatal paradigms in rodent models include psychosocial stressors such as maternal separation (21, 75) or a resident-intruder paradigm for older animals (76).

Zebrafish embryos develop externally, though a successful development is also dependent on the maternal level of cortisol (77), as in mammals. Particularly, developing zebrafish embryos completely rely on maternal depositions of cortisol and its receptor (GR) distributed throughout the ooplasm. The embryo’s HPI axis and associated regulatory systems (including expression of HSD11B) develop during hatching (2–3 days post fertilization, dpf), after which the HPI axis can be triggered (73, 78). Disturbances in the maternal storage of glucocorticoids and their receptors lead to morphological (including neural system) and behavioral alterations. Under laboratory conditions, these disturbances can be simulated by knocking down selected genes or by injecting external glucocorticoids (77, 78). An exemplary stressor of post-hatched larvae and older individuals is a swirl in an experimental tank or chasing with an aquatic net or psychosocial stressors analogical to those used for rodents (79–81).

The stress response in rodents and zebrafish is determined physiologically by measuring glucocorticoid levels or behaviorally by testing the intensity of the startle response or assessing thigmotaxis and boldness in an open field arena or a cylindrical aquarium (80).

The stress reaction in rodents is very similar to that of humans after prenatal development and throughout the whole life (except for the main glucocorticoid). The range of stress paradigms is wide, offering the possibility to model acute or chronic stress, both of a physiological and psychosocial origin. In contrast, stress reaction in prenatally developing zebrafish is determined by completely different mechanisms. Surprisingly, even with the absence of maternal care, the early embryo is still affected by chronic maternal stress. The development of zebrafish is rapid and after three days of life it is possible to observe a response to various stress factors. However, although plenty of distinct stress paradigms have been developed for both models, the behavioral response may require further examination, to not be mistaken for other pathological behavior such as abulia or impairment in social behavior (82).

##### Malnutrition Modeled in Mammals and Zebrafish

The effect of malnutrition on the development of schizophrenia was confirmed after devastating famines in Dutch and Chinese populations during the 20th century, when the risk of developing schizophrenia increased twofold for those whose mothers experienced the famine during pregnancy (83). Today’s knowledge indicates, that some relevant risk factors of schizophrenia can originate in protein deficiencies and imbalances in intake of vitamins or trace elements (84).

To simulate prenatal and perinatal malnutrition, rat dams are usually fed with a diet including approximately 6% of casein, and postnatal protein malnutrition of pups is modeled by approximately 8% of protein in their diet (84). In comparison, the diet of well-nourished developing rats contains between 18 and 25% of proteins (85–87).

Prenatal malnutrition in rodents can result in abnormal brain morphology with lowered volumes of hippocampus, caused by a decreased number of cells and poorer arborisation, and medial prefrontal cortex (PFC), caused by decreased cell size (87). The neurotransmission in proteinally malnourished rodents is abnormal. The basal level of dopamine is increased in PFC (and nucleus accumbens and VTA) and is accompanied by analogies of positive (hyperlocomotion) and negative (lowered preference to sucrose over water) symptoms of schizophrenia in adult animals malnourished during prenatal and early postnatal development. In only prenatally malnourished rats, the basal dopaminergic level is decreased, which can be associated with cognitive impairments similar to these often seen in schizophrenic patients (85, 86).

Another potent risk factor is a low intake of vitamin D, mediating a higher risk of developing schizophrenia in the offspring of mothers living in or moving to high latitude and urban areas. The intake of vitamin D is the lowest at the turn of winter and spring, with the most severe impact on the foetus during the third trimester (3, 8, 67, 88). In rodent models of prenatal vitamin D deficiency, pregnant dams are temporarily fed with a diet lacking vitamin D, but preserving calcium and phosphorus. Even though the diet is restored after delivery, the offspring in adulthood display schizophrenia-like neurotransmission, brain morphology and behavior (3, 8, 67, 89).

Contrary to rodents, there is a relatively low amount of information available on the effect of malnutrition on the development of zebrafish, even though it is obviously significant. Though the external development of zebrafish, maternal nutrition affects embryos *via* the quality of egg yolks. The effect was observed e.g. in relation to excessive maternal intake of selen, resulting in malformations of the whole central nervous system of offspring, a reduced number of successfully hatching offspring, and altered dopaminergic and serotoninergic systems and adult behavior and learning (88).

The risk of developing schizophrenia also increases with an imbalanced intake of other nutrients, such as folate or homocysteine (90).

##### Maternal Immune Activation Modeled in Mammals and Zebrafish

Mammalian foetal neurodevelopment can be threatened by the maternal immune activation of a viral, bacterial, parasitic or even autoimmune origin (27) depending on the genetic susceptibility or previous experience with the antigen of both parents (91, 92). Clinical studies have documented an increased risk of the development of schizophrenia after maternal infection of an influenza virus (93, 94), bacterial infections of the upper respiratory and reproductive tracts (95) or *Toxoplasma gondii* (96, 97).

Poly (I:C) is commonly used as a viral antigen and LPS as a bacterial antigen, both activating microglial and astrocytal production of pro-inflammatory cytokines (IL-1β, TNF-α), which are probably responsible for disruptions in myelination resulting in the reduction of white matter (98). We also documented that early immune activation by LPS in rodents induces persistent alterations in levels of neurotransmitters including their metabolites, activation of the kynurenine pathway of the tryptophan metabolism, and astrogliosis and hippocampal volume reduction (99). In addition, higher blood levels of IL-1β, IL-8, IFN-γ, TNF-α and especially IL-6 were observed following maternal immune activation, suggesting a role of maternal pro-inflammatory cytokines in the neurodevelopment of the fetus and subsequent schizophrenia in adulthood (8, 27).

In contrast, only little is known about the effect of immune activation on neurodevelopment in zebrafish. Although schizophrenia-associated behavior has been induced by immune activation in adult individuals. Increased level of pro-inflammatory cytokines in zebrafish is associated with a set of symptoms altogether known as sickness behavior, suggesting a similar effect of immune activation on behavior as in rodent models. These symptoms include reduced motivation to explore novel object and to spent time near conspecifics in comparison control animals (100).

##### Modeling Aberrant Neurodevelopment Using Brain Lesions in Mammals and Zebrafish

Differences in brain architecture connected to schizophrenia most commonly include enlarged lateral ventricles and reductions in grey and white matter in cortical and subcortical regions, as observed in post-mortem and brain imaging studies. Exact impact of particular aberrantly developed brain structure on development of schizophrenia can be investigated by perinatal brain lesions in neurodevelopmental animal models (18, 101–103). The rough brain ontogenesis and morphology is relatively conserved in vertebrates enabling the involvement of particular brain areas in psychiatric disorders to be studied (104–107). In neonatal rodents, lesions made in the PFC, ventral hippocampus (homological to anterior hippocampus in humans), amygdala or nucleus accumbens resulted in e.g. altered connectivity or neurochemistry of the limbic circuit or altered cytoarchitecture of the PFC, accompanied by schizophrenia-like symptoms, appearing during the adolescence (5, 18, 21, 108–110).

**Figure 1:**
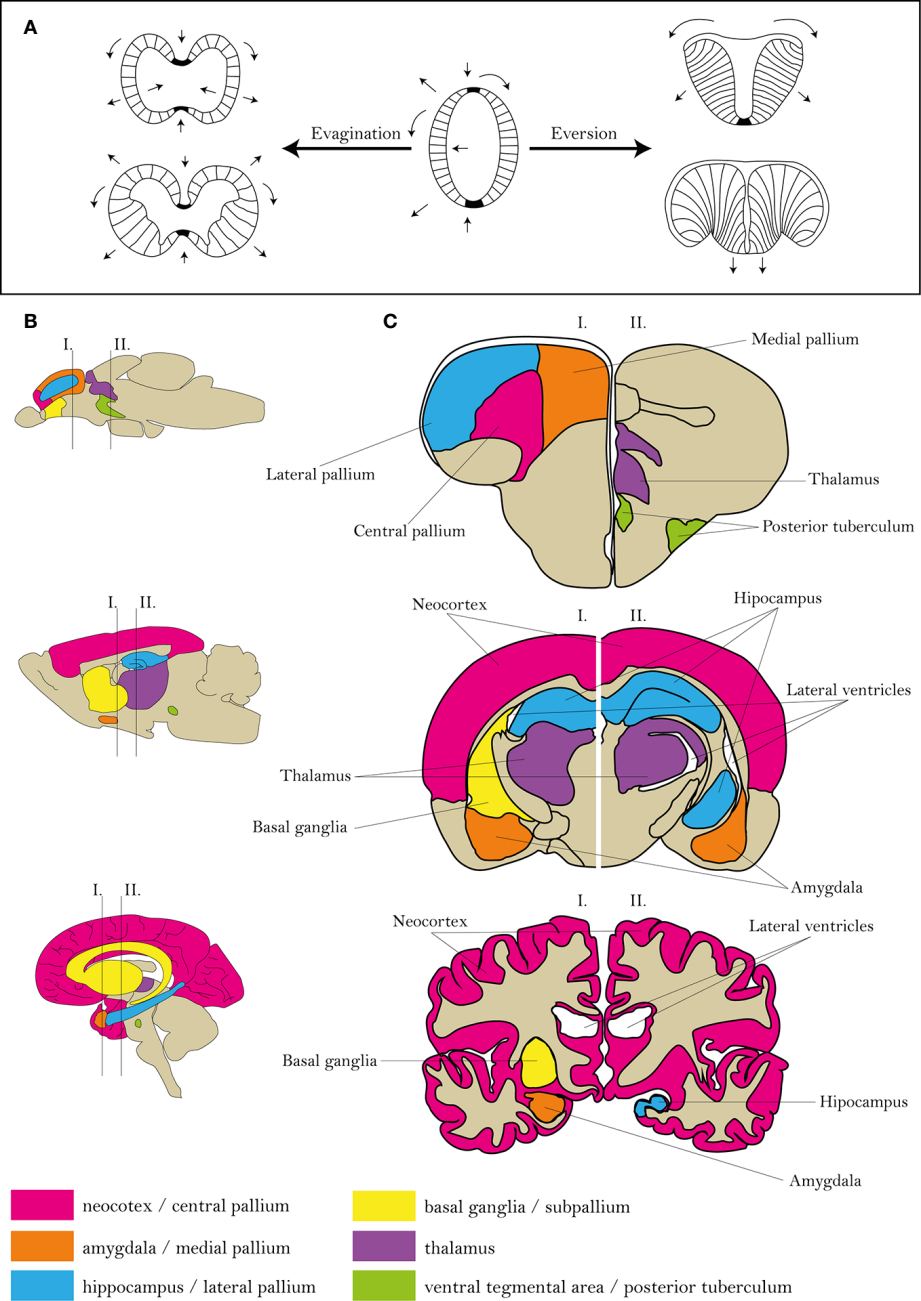
Comparison of zebrafish, rat and human brain morphologies. **(A)** Differences in mammalian and zebrafish CNS ontology. **(B**, **C)** Scheme of human, rat and zebrafish homological brain areas mostly alternated in schizophrenia. Figures were modified from 104–107, 116.

The reasonable utilization of the zebrafish model requires a comparison of actinopterygian and mammalian neural systems (111). Similarly to mammals, most advanced cognitive tasks in fish are processed in the telencephalon and diencephalon (112, 113). However, differences in the ontogenesis of the telencephalon predetermine the distinct morphology and arrangement of individual areas (111, 114). The most probable theory explaining the development of fish telencephalon is the eversion theory (115). A mature zebrafish telencephalon is formed by two hemispheres, similarly to mammalian, although the zebrafish hemispheres are centrally divided and dorsally covered by a large T-shaped ventricles (**Figure 1**). In comparison, as a result of evagination, mammalian hemispheres surround three central ventricles (116, 117).

The zebrafish telencephalon can be functionally divided into the dorsal part (pallium) and ventral part (subpallium). The pallium is further divided into the central, lateral, medial, posterior and ventral parts, probably functionally corresponding to the isocortex, hippocampus, amygdala and piriform cortex of mammals, the subpallium to mammalian sub-cortical regions (111, 112, 116). This arrangement corresponds to that based on expression of genetic markers (111); however, discrepancies between studies can exist based on the sorting criteria (111, 118).

Lesions in both rodent and zebrafish are commonly made by injecting excitotoxic agents, such as ibotenic, kainic or quinolinic acid into the region of interest (108–120).

Even though there are differences between rodents and humans, the general morphology is highly conserved, suggesting a strong construct validity of the models. In contrast, a great deal of effort has been put into determining the homology between the brains of zebrafish and humans; nevertheless, discrepancies still exist and require further analysis (104–107).

### Neurochemical Consequences of Schizophrenia: Pharmacological Models

Pharmacological models are inspired by three sources: neurochemicals and molecular findings in schizophrenia patients, the pharmacodynamic properties of antipsychotics and the observation of psychotomimetic drugs inducing psychotic states in healthy individuals as well as worsening the already existing symptoms in schizophrenic patients (17, 21, 121). The use of psychotomimetic agents in pharmacological modeling has been typically based on glutamatergic and dopaminergic neurochemistry and other (serotonergic) neurotransmitter systems (122). The imbalance of the equilibrium in excitatory and inhibitory neurotransmission often persists for a long time or even permanently (123). Consequences of acute, chronic and early life exposure to psychotomimetic drugs have been intensively tested in animal models (124).

Exposure to psychotomimetic drugs is often combined with other strategies such as neonatal lesions e.g. to test the increased sensitivity to psychotomimetic drugs in adolescence or to strengthen the overall construct validity of the model (125).

The utilization of all mammalian neurotransmitter and neuromodulator systems enables the use of zebrafish pharmacological models, although based on distinct brain morphology some differences between zebrafish and mammalian neurotransmitter systems exist (126). The administration of drugs in rodents, but also in zebrafish is performed by intraperitoneal, intramuscular and subcutaneous injections. In addition, zebrafish can be exposed to drugs non-invasively, *via* inhalation of dissolved drugs in the experimental tank (127).

#### Glutamatergic System in Mammals and Zebrafish

The main vertebrate excitatory neurotransmitter glutamate is highly distributed throughout the brain of mammals as well as zebrafish (128, 129). Glutamate interacts with three families of ionotropic receptors (N-methyl-D-aspartate-type glutamate (NMDA), α-amino-3-hydroxy-5-methyl-4-isoxazole propionic acid receptor (AMPA) and kainate receptors) and three families of metabotropic receptors (mGlu), altogether containing eight subtypes. In relation to schizophrenia, NMDA and AMPA receptors have been studied predominantly. Other candidate targets are metabotropic mGlu1 receptors located on a postsynaptic membrane, which modulates the activity of NMDA receptors, and presynaptic mGlu2/3 receptors, which inhibits the release of glutamate in a negative feedback loop (130). Systemic administration of non-competitive NMDAR antagonists (phencyclidine (PCP), dizocilpine, ketamine) affects glutamatergic and GABAergic neurotransmission as well as its monoaminergic neuromodulation of corticolimbic, corticostriatal and other cortical and subcortical circuits (123). The behavioral changes after acute administration correspond to an acute psychotic state, while changes during chronic administration more likely correspond to chronic schizophrenia and elicit analogs of all, positive, negative and cognitive, symptoms (7, 17, 131). The changes in cortical neurotransmission were confirmed by an *in vivo* magnetic resonance study of the rat brain, after repeated exposure to ketamine (17, 123, 132); non-competitive NMDAR antagonists probably preferably target the parvalbumin GABAergic interneuron, in basal conditions inhibiting excessive firing of pyramidal neurons of the prefrontal cortex in a negative feedback loop (123). Dysfunction of the interneurons results in excitotoxicity and subsequent neurodegeneration in cortical and subcortical areas. This assumption is supported by findings of a lower cortical parvalbumin level in schizophrenia patients pointing to a probable dysfunction of these cells in schizophrenia (17).

Rat models have been recently used to study the connection to other neurotransmittery and neuromodulatory systems complementary to the detailed analysis of the glutamatergic system alone (133, 134). The theory of impaired PFC glutamatergic excitatory and inhibitory control of VTA, which in turn incorrectly regulates striatal dopaminergic activity *via* the mesolimbic pathway resulting in an inadequate regulation of the feedback loop from striatal dopaminergic cells to PFC glutamatergic cells, seems to be especially reliable (133, 135).

#### Dopaminergic System in Mammals and Zebrafish

Dopaminergic theory has long been the major postulated neurochemical theory of schizophrenia and to date still occupies an important position in schizophrenia research (136). Recently, however, it has been discussed more in combination with other theories (glutamatergic theory) than alone, to explain the complex mechanism underlying not only positive, but also negative and cognitive symptoms of schizophrenia (134, 137). Disturbances specifically in dopaminergic neurochemistry and their consequences are modeled using agonists of dopaminergic receptors apomorphine or amphetamine.

Alterations mainly in three of four main dopaminergic pathways of the mammalian brain are related to presence of specific schizophrenia symptoms (4, 138). Overstimulation of mesolimbic pathway has been traditionally assumed to be responsible for positive symptoms, lowered dopaminergic stimulation of the prefrontal cortex for negative and cognitive symptoms and hyperactivity of nigrostriatal pathway for changes in motor activity (139, 140). However, only the positive symptoms have been successfully treated using typical antipsychotics interacting selectively with dopaminergic D_2_ receptors (134).

The dopaminergic system is distinct between zebrafish and mammals. In mammals, the mesocortical pathway projects from the VTA to the PFC, the mesolimbic pathway projects from the VTA to nucleus accumbens and the nigrostriatal pathway projects from the substantia nigra pars compacta to the dorsal striatum (141). In zebrafish, dopaminergic cells have been localized in the diencephalon, hindbrain and subpallium. On the other hand, only catecholaminergic fibers have been detected in the pallium. Interestingly, no dopaminergic cells have been observed in the mesencephalon where three out of four mammalian dopaminergic brain pathways originate (141, 142). Also, midbrain dopaminergic neurons are present in relatively more primitive and evolutionary older cartilagous fish, suggesting a secondary loss during evolution (143). The source of pallial catecholamines may be the diencephalic posterior tuberal nucleus, which has been proposed to be equivalent to the mammalian mesocortical pathway (141).

The zebrafish ventral pallium is assumed to be homological to the mammalian basal ganglia (striatum) and its innervations and projections are suggested to be analogous to the nigrostriatal and mesolimbic pathways (141, 144).

Two families of dopaminergic receptors have been identified in both mammals and zebrafish, a D_1_-like family coupled to G_s_-proteins and a D_2_-like family coupled to G_i_-proteins (**Table 1**) (145).

**Table 1:** Dopaminergic receptors in rodents and zebrafish.

Isoforms of dopaminergic receptors				
Family	D_1_-like	D_2_-like		
Rodents	D_1,_ D_5_	D_2L_, D_2S_	D_3L_, D_3S_	D_4_
Zebrafish	D_1_	D_2a_, D_2b_, D_2c_	D_3_	D_4a_, D_4b_, D_4c_

In rodents the D_1_-like family contains D_1_ and D_5_ receptors and the D_2_-like family contains D_2_, D_3_ and D_4_ receptors. Each of the receptors is encoded by a single gene. In addition, the mRNA of D_2_ and D_3_ is alternatively spliced into two, short (S) and long (L), isoforms (108, 145–147). Rodents express the same number of isoforms as humans, but the number of isoforms differs in zebrafish (148). In zebrafish, only one isoform of D3 and no D_5_ receptor has been identified. In contrast, three isoforms of D_2_ and D_4_ receptors have been identified, at least some of which are encoded by distinct genes (D_2a_, D_2b_) (47, 148, 149).

Elucidation of the homology between mammalian and zebrafish dopaminergic systems by comparing zebrafish and rodent brains is essential for understanding the effects of genetics on neurodevelopmental distributions and strengthening the translational validity of the models (149–151).

#### Serotoninergic System in Mammals and Zebrafish

Serotonin plays a pivotal role in the neuromodulation of glutamatergic, GABAergic, dopaminergic and other systems, modulating, inter alia, the activity of the mesocortical, mesolimbic and nigrostriatal pathways (4), regulating mechanisms of emotions or cognitive functions such as sensorimotor gating or executive functions (152). Suspicion of its role in schizophrenia came primarily from the fact that serotonergic hallucinogens (LSD, mescaline, psilocybine and others) mimic the positive symptoms of an acute psychotic state, and particularly visual hallucinations, but not acoustic hallucinations, which in schizophrenia usually dominate over visual ones (153). On the other hand, eliciting only particular symptoms can be taken as an advantage applicable in studying fundamental neurochemical substrates and mechanisms of schizophrenia, and its specific subtypes or combinations with other psychiatric illnesses (152). Post-mortem studies later revealed alterations in expression of serotonergic receptors and molecules involved in the transport and metabolism of serotonin in cortical and subcortical brain regions, which was confirmed in animal models (136, 154).

To date, a total of seven types of serotoninergic receptors have been identified divided into at least 14 subtypes in humans. Most of the receptors are metabotropic (140). From these is probably mostly alternated expression of 5-HT_1A_, 5-HT_2A_ and 5-HT_2_ subtypes (136). All three receptors are coupled to G-proteins. 5-HT_1A_ is coupled to G_i_/G_o_-proteins, and 5-HT_2A_ and 5-HT_2C_ interact with G_q_-proteins (4, 140, 155).

Accordingly to the UniProt protein database, in mice, rats and humans, the expression of only one isoform of 5-HT_1A_, 5-HT_2A_ and 5-HT_2_ has been identified, while in zebrafish the expression of two isoforms of each of these receptors has been observed^1^ (156), which indicates the necessity to compare the involvement of individual zebrafish isoforms in relevance to schizophrenia.

The sources of serotonin in the mammalian brain are dorsal and two medial raphe nuclei located in the brain stem (and a minor population of serotonergic cells in hypothalamus) (157). The raphe nuclei releases serotonin in the hypothalamus, amygdala, septum, hippocampus, nucleus accumbens and the frontal cortex (140). In comparison, the organization of a zebrafish’s serotonergic system is more complicated. Neurons are located into nine nuclei organized into four complexes, namely the pretectal, paraventricular, rostral raphe and caudal raphe complexes (158). The zebrafish raphe neurons project abundantly into the dorsal telencephalon and more poorly into the ventral telencephalon (151, 158). Both mammalian and zebrafish serotonergic neurons also project into other brain areas including the brain stem and its network, which is responsible for the startle response (157, 158).

Altogether, a detail analysis is required to identify and describe all genetic, morphological and functional differences between zebrafish and mammalian serotonergic system for translational research (158).

#### Other Systems Hypothetically Involved in Schizophrenia

Imbalances in the acetylcholinergic, histaminergic, noradrenergic and endocanabinoid neurotransmitter systems have recently been revealed in relation to schizophrenia. Moreover, schizophrenia symptoms may appear also in reaction to elevated levels of kynurenic acid, a neuroactive metabolite of L-tryptophan interacting with glutamatergic and acetylcholinergic receptors. All of these neurotransmitter systems have been identified in zebrafish (145, 159–161). Similarly to the mammalian brain, nicotinic and muscarinic, H_1_, H_2_ and H_3_ histaminergic, α_2A_, α_2B_, and α_2C_, β_1_ and β_2A_ adrenergic and CB_1_ and CB_2_ cannabinoid receptors have been identified in the zebrafish brain (145, 162, 163).

## Face Validity of Rodent and Zebrafish Models

Analogously to the majority of other mental disorders, face validity of all animal models represents a challenging issue due to the lack of verbal reporting of mental conditions. Hence, face validity evaluations are limited to analyses of behavioral patterns and instrumental neurophysiological approaches such as electrophysiology. Completely restricted is modeling of some positive (verbal hallucinations, disorganized speech), negative (poverty of speech, alogia) and higher cognitive symptoms with speech as a common denominator (**Table 2**).

**Table 2:** Analogs of schizophrenia symptoms in rodent and zebrafish models.

	Schizophrenia symptoms	Rodent	*D. rerio*
**Positive**	Hallucinations, delusions, disorganized thinking	Hyperlocomotion, erratic movement	↑ number of tail bouts and velocity of swimming, erratic movement
	Stereotypic behaviors	Circling, repetitive sniffing	Circling
	Vulnerability to stress	↑ amplitude of startle response, thigmotaxis	↑ amplitude of startle response, bottom dwelling
**Cognitive**	Impaired information gating	↓ prepulse inhibition of a startle response (PPI)	↓ PPI
	Deficits in memory and learning	Prolonged exploration of a familiar object, impaired spatial learning	Prolonged exploration of a familiar object, impaired spatial learning
	Deficits in executive functions	Impairments in latent inhibition and acquisition, performance and capacity of working memory	Impairments in acquisition, performance and capacity of working memory
**Negative**	Anhedonia	↓ preference of appealing place or object (e.g. palatable food)	↓ preference of appealing place or object (e.g. palatable food)
	Abulia	Deficits in motivation to gain reward (palatable food, explored area, interaction with a familiar conspecific)	Deficits in motivation to gain reward (palatable food, explored area, interaction with a familiar conspecific)
	Social withdrawal	↓ social interaction, ↓ time spent close to a conspecific in SPT	↑ interindividual distance in a shoal, ↓ cohesion of the shoal, ↓ time spent close to a conspecific in SPT, ↓ social interactions in MBT

PPI,prepulse inhibition; SPT, social preference test; MBT, mirror biting test. In each test needs to be considered the age of the animal (164). The PPI of startle response is often performed on larval zebrafish and therefore do not correspond to schizophrenia symptoms of adult humans but is rather a nerodevelopmental model studying mechanism of PPI. Another thing to be considered is the particular strain of rodent as well as fish models, whereas these may differ in behavioral traits, such as boldness, sociability or agressivity. Therefore also the suitability of particular strains for individual tasks differs (165, 166). Data were taken and modified from 7, 14, 70, 108, 127, 167–176.

The overall zebrafish and rat behavioral repertoires have been well characterized under natural as well as laboratory conditions (**Table 2**, 168–170, 177, 178). However, a summary of behavioral repertoire is not always sufficient to correctly interpret a behavioral analysis and the overall biorhythm of a model organism often needs to be considered. Zebrafish are diurnal and their activity varies between light and dark phases of the day and is not constant but rather fluctuates during a single phase. Therefore, the results of studies performed in the light can differ from these observed in the dark and can also vary throughout a session (179, 180).

### Positive Symptoms Modeled in Mammals and Zebrafish

#### Hyperlocomotion and Erratic Movement

The analogy between hyperlocomotion in animal models and psychosis has been inspired elicitation by two groups of drugs sharing psychotomimetic properties in humans: agonists of dopaminergic receptors (such as apomorphine) and antagonists of NMDA receptors (MK-801, PCP and ketamine) (171, 181, 182). The underlying mechanism is suggested to be an elevated dopaminergic activity in the mesolimbic pathway (109, 183). In addition, both typical and atypical antipsychotic drugs decrease psychosis in schizophrenia patients as well as hyperactivity in animal models (182, 183).

Locomotion in rodent models is tested in an open field arena, without a ceiling and with high and usually dark walls not allowing the animal to escape. In contrast to healthy animals, which spent a lot of time resting or close to the walls (thigmotaxis), hyperlocomotor rats move a longer distance and frequently cross the center of the arena (184–187).

The locomotor activity of zebrafish is characterized by the velocity of swimming and the distance swum, the continuity of movement, number and speed of swim bouts and standstills, and the direction of swimming and its changes (149). The fast development of zebrafish allows positive symptoms to be studied in relation to early neurodevelopment, when the earliest movement of zebrafish is observable 18 h post fertilization (188).

As mentioned above, the results of experiments performed in light and dark can differ. The effect of the non-selective agonist of dopaminergic receptors apomorphine had no significant effect on the swimming activity of larval zebrafish in visible light. In contrast, in the dark or under infrared lighting, apomorphine induced dose-dependent bi-phasic changes in locomotion in zebrafish as well as rats, when slight hypolocomotion was followed by hyperlocomotion in both, indicating a similar reaction in zebrafish and laboratory rodents in the dark (149, 180).

#### Repetitive Behavior, Stereotypy, and Circling

Common positive symptom of schizophrenia is stereotypy, particularly expressed in repetitive speech (echopraxia, echolalia), repetitive grimacing or mannerisms. In rodents, homological stereotypical behavioral patterns such as repeated sniffing, licking, gnawing and circling were identified (189, 190), which are also commonly seen in zebrafish. Interestingly, in contrast to rodents, circling behavior in zebrafish occurs naturally, accompanying mating and agonistic activities (179). Circling behavior was assessed to be a consequence of an imbalance in dopaminergic neurochemistry between cerebral hemispheres and subcortical regions (particularly the striatum), with preferred direction towards the less dopaminergically active side of the brain. Nevertheless, NMDA receptor antagonists MK-801 and ketamine elicited or amplified circling in zebrafish and rodents with no preferred direction (167, 181, 190–193).

#### Stress Associated Behavioral Response

Schizophrenia patients typically respond vulnerably to stressful situations, expressed as higher reactivity to sudden intensive stimuli, repeated sensory stimuli and higher activity of the HPA axis (194, 195). In animal models, the vulnerability to stress can be examined, testing reactivity to a sudden sensory stimulus or habituation to novelty. Each novel situation can carry a potential danger and animals in a novel environment act with an increased anxiety (expressed as a thigmotaxis) to protect themselves against the anticipated threat. Successively, the anxiety individually decreases during habituation to be replaced by the natural tendency to explore followed by a repertoire of comfort behavior expressed as self-grooming. The relationship between anxiety, exploratory behavior and comfort behavior in rodents is usually examined in an open field arena (184–187).

In comparison, zebrafish individuals in a novel tank at first swim to the bottom, where they move erratically with frequent freezing combined with a higher velocity and often changes in the direction of swimming accompanied by intensive reactivity to stressors, expressed as a C-bend shaped startle response followed by quick avoiding swimming. After the initial anxious phase, individuals start to explore the aquarium at first horizontally and afterwards vertically. Finally, in a resting state, zebrafish individuals swim slowly and forward, occasionally and moderately changing direction. An alternative to a simple tank is a cylindrical tank enabling to more clearly distinguish between vertical parts of the tank (149, 178, 196–199).

In some cases, observing only the spontaneous activity can be misleading, the decreased anxiety can be mistaken for hyperactivity or impaired sociability and the correct interpretation of behavior requires additional tasks. The exploratory arena can be enriched by a novel object to observe behavioral patterns associated with exploration or by conspecifics to test the sociability (82, 184–187), as demonstrated after exposure to ketamine affecting inter-individual distances in shoals, but not any other parameters of social behavior, pointing to reduced anxiety rather than impaired social behavior (192).

Alternatively, in both, rodent and zebrafish models can be tested a reactivity to repeated sudden and intensive acoustic, luminous or tactile stimuli (172, 200). The reactivity to repeated sensory stimuli in schizophrenia patients and pharmacological animal models has been found to be higher, which is associated with impairments in habituation (136).

### Negative Symptoms Modeled in Mammals and Zebrafish

#### Impaired Social Behavior

Probable origins of social impairements occurring in schizophrenia arise from an affective flattening and impairment or inability to distinguish and describe others’ as well as one’s own emotions (5). However, even though probable mechanisms are recently being uncovered, current treatment is insufficient in their normalization. Social dysfunctions still bother a high percentage of people suffering from schizophrenia and often lead to a complete social isolation. Therefore, to model social relationships and the impairements related to schizophrenia are required highly social animal species.

Zebrafish individuals tend to form dynamically changing groups known as shoals varying in the size from few to several hundreds of individuals (201). Properties of each shoal are affected by external as well as internal factors and these needs to be considered in every analyze. External factors affecting shoaling include latitude, water flow, vegetation and presence of predators (201, 202). Internal factors include strain, number, age, size or sex of individual members (12, 13, 165).

Individuals are able to coordinate their movement and polarise the whole shoal into one direction, in behavior known as schooling. Schooling increases the movement of the whole group and probably confuses predators; however, it is disadvantageous during foraging; therefore, groups tend to switch between organized schooling and disorganized shoaling (15). Shoals can be described by average neighbor and farthest neighbor distance, duration of excursions and polarization and tightness of the group (14).

In comparison to zebrafish’ shoals, which seem to be rather socially uniform, rats tend to form socially diverse groups, where each individual has its role. In a natural environment, small hierarchical groups are dominated by a single male and host few females and juveniles, accompanied by similar groups. The dominant male defends the group against intruders by threatening and fighting until the unfamiliar male exhibits submissive behavior or leaves. In urban areas, colonies of hundreds of individuals have been observed, staying nearby to places offering easy foraging. Interestingly, under laboratory conditions, even individual males prefer to form hierarchical groups rather than live alone, which indicates the importance of sociability in the rat’s life (163, 203).

In relation to schizophrenia, fish exposed to MK-801 were significantly less attracted to their conspecifics and spent significantly less time trying to reach a group than control fish, suggesting MK-801 mimics social withdrawal in zebrafish (127). Similarly, rats were less willing to spent time close to their conspecifics after exposure to MK-801 and, on the contrary, the dopaminergic agonist amphetamine failed to affect social behavior (and thus elicit analogies of negative symptoms) in accordance with the general glutamatergic and dopaminergic theory of schizophrenia (204).

Social behavior has been studied also in other rodent as well as fish species. As an alternative rodent model of psychiatric diseases has been recently suggested the prairie vole (*Microtus ochrogaster*), displaying highly complex social behavior including social bonding and parenatal behavior (205).

In fish species, such as three-spined stickleback (*Gasterosteus aculeatus*) have been observed variances in personal traits including boldness and aggressivity, affecting social bonding and other interindividual relationships (206). As we and others observed, the personal traits of social fish individuals origin in genetics, interindividual relationships and interspecies relationships or environmental factors during the early life as well as during the adulthood (206–210).

#### Anhedonia

Anhedonia is defined as a reduced ability or complete disability to experience pleasure, ranked by several standardized psychometric scales in humans (Scale for the Assessment of Negative Symptoms (SANS), the Schedule for the Deficit Syndrome (SDS), the Positive and Negative Syndrome Scale (PANSS), and the Scale for Emotional Blunting (SEB)) and is traditionally marked as one of the negative symptoms of schizophrenia (211). Several recent studies did not confirm any lack of experiencing pleasure in schizophrenia in real time, proposing the loss of interest in pleasurable activities to originate from the altered retrieval of memories of hedonic emotions, related to dysfunctions in working memory essential for anticipation of pleasure and interest in pleasurable activities in the future, instead of a disability to experience pleasure itself (212, 213).

In rodents, anhedonia is tested by paradigms challenging sensitivity to rewards related to motivation to gain a reward. The most frequently used paradigm to test anhedonia in rodents is the sucrose preference test examining preference towards a sweet liquid (containing sucrose or saccharin) over clear water, as healthy animals do. Another paradigm is the conditioned place preference test combining anhedonia and associative learning, whereby an attractive object (palatable food, drug, conspecific) is placed in a chosen place to test the preference of such a place. Sensitivity to a reward can be directly tested invasively, by implanting a stimulating electrode to a brain area activating the reward circuit. The animal is simultaneously trained to activate an electrode to stimulate the reward circuit by itself with mild electric pulses. The frequency and willingness of the self-stimulation are evaluated (173, 214).

In zebrafish, anhedonia is determined analogically to rodents by decreased motivation to gain a reward based on the activity in a conditioned place preference test and hypophagy (215).

#### Deficits in Motivation to Gain a Reward

In animal models tested by a reaction to successively increasing a reward, e.g. sucrose in a diet, healthy animals react with increased licking. In contrast, mice with genetically increased dopaminergic activity in the striatum (accordingly to schizophrenia patients) are less active in gaining an increasing reward (216). An alternative reward is a conspecific, e.g. in a social conditioned place preference test, in which a healthy individual is attracted to exploring places previously hosting a familiar conspecific and the attractivity. In contrast, animals exposed to PCP were less attracted to places previously hosting a conspecific and were generally hypoactive (125, 217). Sociability as a reward has also been used in zebrafish models relevant for schizophrenia (218). Alternatively, in rodents the latency of immobility in a water pool can be tested in a forced swim test, a non-specific behavioral task often also used in modeling depression (219).

Comparing behavior of wild fish and fish reared in hatchery, we have shown that the motivation and ability to gain a reward in fish is highly affected by the early-life environment of the individual, indicating some options in fish neurodevelopmental modeling and pointing out the importance of the source of laboratory animals (208).

### Cognitive Deficit and Aberrant Information Processing Modeled in Mammals and Zebrafish

Cognitive deficits of schizophrenic patients affect the functional capacity reflected in social life and independence of residential functioning and working (220). The deficits are uncovered in tasks challenging sensorimotor gating (221), working memory (222), attention (223), as well as non-associative (224) and associative learning (225).

#### Aberrant Information Processing: Decreased Prepulse Inhibition of the Startle Response

Sensorimotor gating together with habituation is considered to filter out unnecessary information and disruption of these phenomena is tightly bound to attentional deficits often seen in schizophrenia. Abnormalities in sensorimotor gating are studied by the startle reaction to sensory stimulus and its inhibition (PPI). The principle of PPI consists in an inhibition of a startle reaction by previously applied milder stimulus of the same modality. Disruptions in the PPI have been observed in schizophrenia and other disorders, when the previous mild stimulus does not effectively inhibit subsequent stronger stimulus and the patient reacts with the same (or even higher) intensity than a healthy individual (224, 226, 227).

The neural coding of a startle response and its PPI is remarkably more complex in rodents than in zebrafish, suggesting that the rodent neurobiological substrate corresponds more to humans. Nevertheless, as in mammals (174), PPI in zebrafish can be disrupted in genetic, pharmacological and other models relevant for schizophrenia, indicating that PPI represents an evolutionary conserved mechanism involved in sensorimotor gating. Therefore, zebrafish model is still applicable in studying basic mechanisms of sensorimotor gating and its association with psychiatric disorders (228). Moreover, in zebrafish it is possible to study PPI and its regulation by psychotomimetic drugs from early developmental stage (the 5–6 dpf). Therefore, larval zebrafish offer an insight into effect of early life disruptions and abnormalities of neurochemistry on the developing sensorimotor gating and development of sensorimotor gating itself (172, 228, 229).

In mammals, the whole process is encoded on three levels, mediating the startle reaction, mediating the PPI and modulating the PPI. The startle response and its PPI are mediated by brain stem nuclei including the caudal pontine reticular, cochlear, pedunculopontine, laterodorsal tegmental nucleus, colliculi superior and inferior, and substantia nigra. Additionally, PPI is modulated by cortico-striato-pallido-pontine and cortico-striato-pallido-thalamic circuitry (230, 231). Rat neonatal and adolescent lesion models document the involvement of cortical and subcortical areas in sensorimotor gating. Adult rats after neonatal as well as adolescent lesions of the amygdala react more intensively to sudden sensory stimuli than control individuals. However, in the relevant study, PPI was disrupted only in neonatally lesioned individuals, indicating no direct encoding of the PPI by amygdala but associated structures, such as the basal ganglia or PFC (109).

In comparison, the missing corticospinal connection in zebrafish probably restricts the whole encoding of PPI in zebrafish to the brain stem reticulospinal network (228, 229, 232). A weaker prestimulus affects the probability of PPI and a startle response inhibited by a previous weaker stimulus consists in a slower reaction, but the C-bend magnitude, angular velocity and duration remain unaltered. In comparison, in mammals, a prestimulus also affects the magnitude of a startle response, which is probably given by more complex neural circuitry encoding the startle response in mammals than in zebrafish (228).

In accordance with the hypothesis of a highly conserved evolutionary mechanism, impairments in PPI or the startle response can be normalized by antipsychotic drugs in rodents as well as in zebrafish (200, 228).

#### Learning Deficits: Non-Associative and Associative Learning

Non-associative learning consists in habituation, dishabituation and sensitisation to a sensory stimulus, object or environment (224, 226). Disruptions in habituation in schizophrenia patients were revealed by cognitive tasks studying human habituation based on the startle reaction to a repeated sensory stimulus and comparison of reaction to novelty and familiarity and confirmed by neuroimaging studies (224).

Zebrafish and rodents display rapid (up to 15 min) short- (up to 1 h) and long-term habituation (up to 18–24 h) to repeated sensory stimuli (140, 233–235), after which the responsiveness normalizes. All forms of habituation can be disrupted by NMDA antagonists, depending on the dosage (172, 235, 236).

The classical conditioning consists in association of a sensory reaction elicited by the unconditional stimulus without any need of learning with another unrelated conditional sensory stimulus. Stimuli eliciting strong attraction (reward) or aversion (punishment) are usually used as unconditional stimuli. Impairment of associative learning in schizophrenic patients has been studied e.g. in tasks pairing familiar objects with places in a space. Distributions of the glutamatergic system resulting in decreased NMDA activity in hippocampal areas may be responsible for impairments of associative learning in schizophrenia (237).

Zebrafish and rodents are teachable in both classical and operant conditioning learning tasks. The classical conditioning of rodents can be performed in various mazes including a radial maze or a Barnes maze (236, 238), and in the case of zebrafish in a Y or plus maze (199, 239, 240).

In zebrafish and rodent models, conspecifics can be used as a reward. Social behavior in zebrafish is predominantly based on the fear of predators and foraging (11, 15). In spatial learning tasks, a tested individual is highly attracted to the arm of a maze containing a conspecific or group of conspecifics, and the individual tends to stay in the arm even after removing the conspecifics (199, 239). Palatable food can also be used as a reward for both zebrafish and rodents and, on the contrary, a mild electric shock can be used as an aversive stimulus (175). The operant conditioning of zebrafish was demonstrated by pairing a light impulse with an electric shock in a shuttle box. After learning, individuals moved to another room after exposure to a light signal to actively avoid the anticipated electric shock, similarly to rodents (199, 241).

#### Deficits in Executive Functions

Executive functions comprise a set of cognitive abilities essential for an individual’s voluntary or adaptive action including attention and working memory, both frequently impaired in schizophrenia (223, 242, 243). Attentional deficits are determined in latent inhibition (LI), continuous performance test (CPT) or 5-choice serial reaction time task (5-CSRTT), intradimensional–extradimensional (ID-ED) paradigms (244–247). Working memory can be tested in spatial tasks, in humans in the Visual Memory Span test (242). Animals are tested for acquisition, performance and capacity impairments, challenged in rodents in a delayed non-matched to place T-maze, Y-maze or radial-arm maze (248, 249). Studies on rodents shown, that impairements in executive functions has developed in pharmacological models, by directly affecting the neurotransmission (176, 247) as well as in genetic models (250) or in neurodevelopmental models, including immune activation in early developmental stage (251, 252). In zebrafish can be executive functions studied also in behavioral studies, observing decision making in Y- or T-maze or in the one-trial inhibitory avoidance test (240, 253) or in brain-imaging studies, in which can be utilized the transparency of zebrafish larvae to track the involvement of neuronal circuits in implementing individual executive functions (254).

## Predictive Validity of Rodent and Zebrafish Models

Since the mid-20th century, two generations of antipsychotic drugs have been developed (4, 255). The first generation of antipsychotic drugs (typical antipsychotics, e.g. haloperidol) was designed as antagonists of dopaminergic D_2_ receptors. However, a simple blockade of D_2_ elicits severe side effects, including parkinsonism or hyperprolactinemia and effectively reverses only positive symptoms. The second generation of antipsychotics (atypical antipsychotics) was designated to more effectively treat negative and cognitive symptoms and the efficiency consists in a lower affinity to dopaminergic receptors and also moderate affinity to serotoninergic, histaminergic, muscarinic and α-adrenergic ones, requiring of these receptors to be expressed in model organisms (4). Second generation antipsychotics are divided into four groups based on their affinity to particular receptors. Parcial dopamine D_2_/D_3_ agonists include aripiprazole and cariprazine, selective antagonists of D_2_ and D_3_ receptors include sulpiride or remoxipride, serotonine–dopamine antagonists (SDA) interact prominently with 5-HT_2A_ and D_2_ receptors and include risperidone or sertindole, multi-acting receptor-targeted antipsychotics (MARTA) interact with a high affinity to several dopaminergic (D_2_, D_4_), serotonergic (5-HT_1A_, 5-HT_2A,_ 5-HT_2c_) as well as histaminergic, cholinergic and other receptors and include clozapine or olanzapine (4, 256). However, second generation antipsychotics can cause extrapyramidal side effects, agranulocytosis or hyperprolactinemia, which is currently leading to the development of the new generation of antipsychotics (4, 255).

The predictive validity of animal models of schizophrenia is based on the potential to normalize positive, negative and cognitive symptoms mirroring the profile of clinical effects in patients. In general, comparing to rodent models, the normalizing effect of antipsychotic drugs in zebrafish models is less intensively studied (**Table 3**). Congruently with the clinical experience in patients, the normalizing effect of antipsychotic treatment is often dose dependent and may be distinct between individual animal models.

**Table 3:** Effects of first (haloperidol) and second (typical representatives) generation antipsychotics on the ability to normalize the schizophrenia-like symptoms modeled in rodents and fish.

Antipsychotics Symptoms		Typical			Atypical												
		Hal			Ari		Sul		Ris			Clo			Ola		
Positive	Hyperlocomotion, erratic movement/↑ number of tail bouts and velocity of swimming, erratic movement	+ −		+	+	ND	+	+	+		ND	+		+	+		+
		P N M G		P	P		P	P	N			P G N		P	P		P
	Circling, repetitive sniffing	+		ND	+	ND	+	ND	+		ND	+ −		ND	+		ND
		G P			P		P		P			P			P		
	↑ amplitude of a startle response, stress-induced thigmotaxis/bottom dwelling	−		−	+	ND	−	−	+ −		+	+		ND	−		−
		P		P	P		P	P	P		S	P			P		P
Negative	Anhedonia	−	−	ND	+	ND	ND	ND	+		ND	+		+	+		ND
		S	P		S				S			P		S	S		
	Deficits in motivation to gain a reward (palatable food, explored area, interaction with a familiar conspecific, safety)	−		ND	+	ND	+	ND	+		ND	+		ND	+		ND
		P			S		P		P			P			P S		
	↓ social interaction (social withdrawal), ↑interindividual distance in a group	−		−	+	ND	+	+	+	−	ND	+	−	−	+		+
		P		P	P		P	P	P	P N		P G	P N	P	P		P
Cognitive	↓ PPI	+ −		+	+ −	ND	−	ND	−	+	ND	+		ND	+		ND
		P G		P	P		P		P G	P N		P G			P		
	Prolonged exploration of a familiar object or conspecific, impaired spatial learning	−		−	+	ND	+ −	+	+		ND	+		ND	+	−	+
		P S		P	S		P	P	P			P			P	P S	P
	Impairments in LI, CPT or 5-CSRTT	−		ND	+	ND	+	ND	+	−	ND	+		ND	+		ND
		N P			P		N		N	P		G			N		

Rodent = yellow, zebrafish = blue; + = normalization, − = no normalizing effect, ND, no data; Hal, haloperidol; Ari, aripiprazole; Sul, sulpiride; Ris, risperidone; Clo, clozapine; Ola, olanzapine; models: P = pharmacological model, N = lesion neurodevelopmental model, M = malnutrition model, G = genetic model, S = stress model, PPI = prepulse inhibition of startle response, LI = latent inhibition, CPT = continuous performance test, 5-choice serial reaction time task = 5-CSRTT. Data taken from 76, 81, 127, 200, 219, 228, 244, 247, 249, 257–288.

Some of the symptoms are treatable by typical as well as atypical antipsychotic drugs with a relatively high efficiency. The typical example are positive psychotic symptoms in humans and hyperlocomotion in animal models while the efficiency of antipsychotics in other symptoms varies, such as in the impaired PPI (127, 200, 228, 249, 257–260, 278, 279). The efficiency to ameliorate the modeled symptoms also differs within the groups of partial agonists of D_2_/D_3_ receptors, selective D_2_ and D_3_ receptor antagonists, SDA and MARTA (247). Moreover, one antipsychotic drug can elicit distinct or even contradictory reactions. Examples are reactions of rodent pharmacological models to clozapine. PCP induced social withdrawal was in one case successfully reduced (261) when in other increased (262) by clozapine. Possible reasons for contradictory reactions to the same antipsychotic drug can be the dose of the drug, the length of a threatening period or the genetic background of the model organism (rats vs. mice). Distinctive traits of individual models leading to contradictory results may be usable in examining pharmacological functions of antipsychotics, but also other drugs used in psychiatry. Known impact on neurochemistry therefore lead to more specific treatment suitable for individual patient’s needs. In such way was observed a normalizing effect of originally anxiolytic drug buspirone on analogs of positive, negative and most importantly cognitive symptoms in mouse genetic model lacking dopamine D_3_ receptors and afterwards in schizophrenia patients using atypical antipsychotic drugs. These observations indicate, that the buspirone works as a 5-HT_1A_ receptor agonist and D_3_ receptor antagonist and therefore may be suitable for treating some cases of schizophrenia (289, 290).

Moreover, contradictory results in individual models contributing to the knowledge of pharmacological function of drugs show a mutually affecting relationship between development of antipsychotic drugs and animal modeling, when animal models are necessary to test pharmacological functions of newly developed antipsychotic drugs and simultaneously antipsychotics are usable to test overall validity of existing animal models and their improvement or to search and develop new animal models.

Hallucinations in schizophrenia patients are often treatable by typical antipsychotic drugs, although atypical antipsychotics are more effective. Occasionally, hallucinations persist treatment even by some MARTA antipsychotics (e.g. olanzapine, quetiapine) and, therefore, these need to be replaced by the most effective of the same group, clozapine (291, 292). In comparison, overall hyperlocomotion and erratic movements have been successfully treated by all of the mentioned antipsychotic drugs in rodents as well as in zebrafish, however not in all cases. Locomotor activity can be affected by agonists of dopamine receptors and can be induced by agonists of both D_1_ and D_2_ receptors or more specifically by drugs binding to only one type. A non-selective agonist apomorphine binds to both types of receptors, alhough with a higher affinity to D_2_ and selective activation of D_2_ can have a stronger impact on locomotor activity in some strains than selective activation of D1, which may suggest a higher impact of D_2_ on locomotion. However in some mutant animals selective agonists of D_2_ do not have an effect on locomotion (166) and insterestingly, activation of D1 receptors can induce hyperlocomotion without other set of common symptoms, stereotypy, suggesting a specific tool in reseach as well as therapy (293). Both dopamine receptors are highly involved in schizophrenia, however, hyperlocomotion in animal models can be also triggered directly *via* NMDA antagonists, which was confirmed on dopamine-deficient mice lacking tyrosine hydroxylase. In this model also haloperidol did not treat PCP or MK-801 induced hyperlocomotion (263). Hence, we assume that confirmation of the analogy between psychosis and hyperlocomotion requires further investigation, including comparison of distinct strains of model organisms (166).

The stereotypical behavior has been shown to be successfully normalized in almost all rodent models by both typical and atypical antipsychotics (264, 265, 280) except for a case of non-significant finding in the rat pharmacological model treated by clozapine (266). We have not found any similar work in zebrafish model, although the stereotypical swimming has been elicited e.g. by MK-801 or by mutations in the dopaminergic system (294, 295). However, an investigation of the normalizing effects of antipsychotic drugs on the stereotypical behavior of zebrafish can deepen the insight into the neurobiological substrate of stereotypical behavior (179). A possible mechanism of stereotypies occurring in schizophrenia suggests a rat genetic model of deleted dopamine transporter, as these animals display stereotypical behavior during eating (43).

The hyperactivity of the HPA axis of schizophrenia patients is normalized by long-term regular usage of both typical and atypical antipsychotics (195). The efficiency of antipsychotics on the increased vulnerability to stress in animal models is documented more sporadically in comparison to the previously mentioned positive symptoms (200). In stressed zebrafish, risperidone reduced bottom dwelling and the blood level of cortisol, whereas in rats, risperidone, clozapine and olanzapine reduced spontaneous startle reactivity, all indicating a reduction in a stress reactivity (81, 200). Though, the reduction in stress reactivity in rats is independent on the exposure to the psychotomimetic drug MK-801 (200), therefore it does not indicate a normalization of a schizophrenia-like symptom. We have not found any study investigating the effect of antipsychotics on stressed zebrafish pretreated by psychotomimetic drugs such as MK-801.

The impaired PPI in schizophrenia patients has been effectively normalized by atypical but not typical antipsychotics (226). In comparison, in animal models, impairments in PPI have been successfully ameliorated by typical antipsychotics as well. An example is chronic treatment or high doses of haloperidol normalizing impaired PPI, as seen in rats as well as zebrafish models (228, 281). It indicates that impaired PPI may be at least partially linked to neurobiological mechanisms underlying positive symptoms and the relation between positive symptoms and impaired PPI has been previously discussed (296, 297). The interconnection between positive symptoms and impairements in PPI are supported by the fact, that both can be modulated *via* dopaminergic receptors (40, 166).

Importantly, atypical antipsychotic drugs do not always reverse all cognitive and negative symptoms in schizophrenia patients (298), as well as in some animal models (282). In example, sulpiride (as a selective antagonist of D_2_ and D_3_ receptors) did not reverse MK-801 disrupted PPI in rats (278) and risperidone (as SDA) did not normalize PCP induced impairments in learning and executive functions by subchronic administration of PCP in rats (244), when in contrary the cognitive deficits are often ameliorated by clozapine and olanzapine (MARTA antipsychotics) (200, 259, 267–272, 283–285). On the other hand, risperidone normalized some cognitive symptoms induced by MK-801 and by hippocampal lesions (249, 273), whereas sulpiride normalized cognitive symptoms induced by metamphetamine (274). Partial dopamine receptor agonist aripiprazole interacting as agonist with D_2_ and 5-HT_1A_ and as antagonist with 5-HT_2A_ receptors has been shown to normalize cognitive symptoms (275, 276) and act neuroprotectivelly *via* reduction of apoptosis in PFC, even when administered repeatedly. In contrary to that, eventhough haloperidol may normalize some cognitive symptoms, its repeated administration supports apoptosis in PFC (299). Some cognitive as well as negative symptoms have been also successfully treated by administration of an alkaloid dehydrocorybulbine binding with a high affinity to σ1 and 2 receptors and 5-HT_7_ receptor (300).

Treatment of negative symptoms in schizophrenia patients with antipsychotics of both generations is generally under discussion, when even MARTA antipsychotics do not always reverse negative symptoms effectively (301). We have not found any work investigating the effect of antipsychotics on anhedonia or abulia in zebrafish models of schizophrenia, but the analysis of the efficiency to normalize impairements in social interactions by typical and atypical antipsychotics in the pharmacological model indicates zebrafish to be a promising model of negative symptoms (127).

Anhedonia and avolition in pharmacological and stress-based rodent models were normalized by atypical, but not by typical antipsychotic drugs (277, 286, 287). Haloperidol has a mildly protective effect against anhedonia when administered before exposure to a stressful situation, although as a treatment it was shown to be ineffective (288). In comparison, olanzapine not only prevents, but also reduces anhedonia in a stress-based rodent model (288). In a rat chronical stress model aripiprazole restored the motivation to gain a revard, however it did not normalize a motivation to escape from a harmful stimulus. On the molecular level aripiprazole affect intracellular dopaminergic signaling in nucleus accumbens (302).

Progressive abulia and anhedonia together result in social withdrawal, the most severe negative symptom crucially worsening the quality of the life of a schizophrenia patient (301). In both rodents and zebrafish models, haloperidol fails to normalize social withdrawal evaluated as decreased social interactions and decreased tendency to stay close to conspecifics, whereas treating with atypical antipsychotic drugs is more often successful (261, 283). Modeling the impairments in social behavior is currently restricted to observation of alterations in behavioral patterns, as modeling the verbal component, which simultaneously interconnects all, positive, negative and cognitive symptoms, is currently impossible.

In summary, the normalizing effect of antipsychotic drugs on various dimensions of schizophrenia-like symptoms has been mapped more systematically in rodents than in zebrafish. Zebrafish models are more likely used to deepen the knowledge of schizophrenia pathogenesis within the context of neurodevelopmental mechanisms and the impact on less complex behavioral markers. Moreover, zebrafish models are often used to study the toxicity of psychotomimetic as well as antipsychotic drugs (10, 303). The promising predictive validity of zebrafish models has been demonstrated in studies focusing on the normalizing effect of antipsychotic drugs on hyperlocomotion, acoustic startle response and its PPI and especially social behavior. Investigating the normalizing effect on other symptoms in zebrafish such as stereotypical behavior can bring relevant information useful in e.g. studying the mechanisms underlying evolutionary conserved behavior affected in schizophrenia, strengthening the translational validity of individual models and subsequently in developing more effective treatments.

## Conclusions and Future Directions

Both rodents and zebrafish play an eminent role in schizophrenia research. Similarities between rodents and human physiology and brain morphology determine the high construct validity of rodent models of schizophrenia. In comparison, differences in zebrafish neurodevelopment result in distinct arrangement of the brain requiring deeper insight to clearly postulate the homology between individual human and zebrafish areas and to determine the construct validity of zebrafish models. Currently, zebrafish are usable primarily to study the effects of genetic and early life factors on the general mechanisms underlying basic neurodevelopmental principles in the field of research drugs and development, and the effects on simple and highly evolutionary conserved behavior.

Despite the widely-known rodent and zebrafish limited behavioral repertoires giving rise to various behavioral tasks, the results of behavioral tasks are often not sufficiently specific for schizophrenia. Concretly, modeling of certain specific language-based symptoms such as verbal hallucinations, disorganized speech and delusions is completely restricted and further novel approaches are required.

To date, the predictive validity has been more systematically studied in rodent than in zebrafish models. Nevertheless, the normalizing effect of antipsychotic drugs on positive, negative and cognitive symptoms indicates a promising predictive validity of the zebrafish model, as well.

To some extent, the limitations of both models could be remediated by their combination and in combination with other research methodologies. Concretely, the complex rodent behavior can be complemented of studying the impact of genetic effects on evolutionary conserved neurobiological substrates in zebrafish and *vice versa*. The genetic screen of zebrafish can uncover mutations altering their development, which can be subsequently studied in rodents in more detail. Subsequently, the known rodent brain morphology and its neurochemistry and the fact that the fundamental morphology and neurochemistry is conserved in vertebrates can be used to identify homologies between zebrafish and mammalian brains. This could strengthen the construct, face and predictive validity of both model organisms.

Therefore, given the fact that the expression of schizophrenia is typically verbal (e.g. verbal acoustic hallucinations and disorganized speech in positive symptoms, or alogia in negative ones), the absence of the possibility to evaluate communication aberrancies represents the major pitfall of the current approaches to schizophrenia modeling and a search for more suitable model organisms possessing highly developed communication abilities (as seen in birds or social insects) is required.

Additionally, the research on the rodent models and the zebrafish model suggests that although the clinical manifestation of schizophrenia can be diagnosed in humans only, the neurobiological substrate for the development of schizophrenia symptoms is apparently present in lower vertebrates. Considering this fully, the fascinating possibility of studying the neurobiological substrate of schizophrenia in evolutionary and developmental contexts opens up.

## Author’s Note

Each of the authors contributed to the manuscript with the knowledge of their own field of interest. VL received Bachelor degree in the field of Molecular Biology and Biochemistry of organisms and the Master of Science degree in Animal Physiology and is currently studying Neurosciences under the supervision of Prof. MUDr. Jiří Horáček, Ph.D., FCMA. KV research interests include the neurobiology of schizophrenia, with a focus in preclinical drug research and development. He is currently a leader of the different projects regarding drug development and study of the novel animal models of schizophrenia. PH obtained PhD degree in Ecology. She worked as a Research Scientists at T. G. Masaryk Water Research Institute at the Department of Applied Ecology, she was focused on community structure and behavior of fish in running waters. She is currently an Assistant Professor at the Institute for Environmental Studies, Charles University in Prague, where she specializes on behavioral ecology of fish. She is a Lecturer in Limnology. JH holds a degree in psychiatry and psychotherapy. His research activities involve the use of brain imaging in the fields of schizophrenia, depression and OCD, psychiatric genetics and the animal modeling of mental disorders

## Author Contributions

All authors listed have made substantial, direct, and intellectual contribution to the work and approved it for publication.

## Funding

This work was supported by the grants GACR 304/18-09296S and 17-04047S, AZV 17-30833A and NV18-04-00260. Institutional support for NIMH-CZ was provided by the project “Sustainability for the National Institute of Mental Health” number LO161 and PharmaBrain” CZ.02.1.01/0.0/0.0/16_025/0007444.

## Conflict of Interest

The authors declare that the research was conducted in the absence of any commercial or financial relationships that could be construed as a potential conflict of interest.

## Footnotes

1 www.uniprot.org/uniprot/?query=5-ht&sort=score

## References

[1] Bhugra D The global prevalence of schizophrenia. PloS Med (2005) 2:e151. 15916460 10.1371/journal.pmed.0020151 PMC1140960

[2] Kinney DK Teixeira P Hsu D Napoleon SC Crowley DJ Miller A Relation of schizophrenia prevalence to latitude, climate, fish consumption, infant mortality, and skin color: a role for prenatal vitamin D deficiency and infections? Schizophr Bull (2009) 35:582–95. 10.1093/schbul/sbp023 PMC2669590 19357239

[3] Samões B Silveira C The role of vitamin D in the pathophysiology of schizophrenia. Neuropsychiatry (2017) 7:362–9.

[4] Horacek J Bubenikova-Valesova V Kopecek M Palenicek T Dockery C Mohr P Mechanism of action of atypical antipsychotic drugs and the neurobiology of schizophrenia. CNS Drugs (2006) 20:389–409. 16696579 10.2165/00023210-200620050-00004

[5] Aleman A Kahn RS Strange feelings: do amygdala abnormalities dysregulate the emotional brain in schizophrenia. Prog Neurobiol (2005) 77:283–98. 10.1016/j.pneurobio.2005.11.005 16352388

[6] Siskind D Siskind V Kisely S Clozapine response rates among people with treatment-resistant schizophrenia: data from a systematic review and meta-analysis. Can J Psychiat (2017) 62:772–7. 10.1177/0706743717718167 PMC5697625 28655284

[7] Bubeníková-Valešová V Horáček J Vrajová M Höschl C Models of schizophrenia in humans and animals based on inhibition of NMDA receptors. Neurosci Biobehav Rev (2008) 32:1014–23. 10.1016/j.neubiorev.2008.03.012 18471877

[8] Meyer U Feldon J Epidemiology-driven neurodevelopmental animal models of schizophrenia. Prog Neurobiol (2010) 90:285–326. 19857543 10.1016/j.pneurobio.2009.10.018

[9] Tordjman S Drapier D Bonnot O Graignic R Fortes S Cohen D Animal models relevant to schiziophrenia and autism: validity and limitations. Behav Genet (2007) 37:61–78. 17160702 10.1007/s10519-006-9120-5

[10] Akande MG Örn S Norrgren L Evaluation of the toxic effects of clozapine in zebra fish (*Danio rerio*) embryos with the fish embryo toxicity test. Int J Pharm BioMed Res (2010) 1:90–4.

[11] Guo S Linking genes to brain, behavior and neurological diseases: what can we learn from zebrafish? Genes Brain Behav (2004) 3:63–74. 15005714 10.1046/j.1601-183x.2003.00053.x

[12] Spence ROW Smith C Male territoriality mediates density and sex ratio effects on oviposition in the zebrafish, Danio rerio. Anim Behav (2005) 69:1317–23.

[13] Buske C Gerlai R Shoaling develops with age in Zebrafish (Danio rerio). Prog Neuropsychopharmacol Biol Psychiatry (2011) 35:1409–15. 10.1016/j.pnpbp.2010.09.003 PMC3021101 20837077

[14] Pham M Raymond J Hester J Kyzar E Gaikwad S Bruce I Assessing social behavior phenotyps in adult zebrafish: shoaling, social preference, and mirror biting tests. Zebrafish Protoc Neurobehav Res (2012) 66:231–46.

[15] Miller N Gerlai R From schooling to shoaling: patterns of collective motion in zebrafish (*Danio rerio*). PloS One (2012) 7:e48865. 10.1371/journal.pone.0048865 23166599 PMC3498229

[16] Volgin AD Yakovlev OA Demin KA de Abreu MS Alekseeva PA Friend AJ Zebrafish models for personalized psychiatry: insights from individual, strain and sex differences, and modeling gene x environment interactions. J Neurosci Res (2019) 97:402–13. 10.1002/jnr.24337 30320468

[17] Morris BJ Cochran SM Pratt JA PCP: from pharmacology to modelling schizophrenia. Curr Opin Pharmacol (2005) 5:101–6. 10.1016/j.coph.2004.08.008 15661633

[18] Benes FM Amygdalocortical circuitry in schizophrenia: from circuits to molecules. Neuropsychopharmacology (2010) 35:239–57. 10.1038/npp.2009.116 PMC3055447 19727065

[19] Morris RW Sparks A Mitchell PB Weickert CS Green MJ Lack of cortico-limbic coupling in bipolar disorder and schizophrenia during emotion regulation. Transl Psychiatry (2012) 2:e90. 10.1038/tp.2012.16 22832855 PMC3309531

[20] Horacek J Mikolas P Tintera J Novak T Palenicek T Brunovsky M Sad mood induction has an opposite effect on amygdala response to emotional stimuli in euthymic patients with bipolar disorder and healthy controls. J Psychiatry Neurosci (2015) 40:134–42. 10.1503/jpn.140044 PMC4354819 25703646

[21] Flores G Morales-Medina JC Diaz A Neuronal and brain morphological changes in animal models of schizophrenia. Behav Brain Res (2016) 301:190–203. 26738967 10.1016/j.bbr.2015.12.034

[22] Li Z Chen J Yu H He L Xu Y Zhang D Genome-wide association analysis identifies 30 new susceptibility loci for schizophrenia. Nat Genet (2017) 49:1576–83. 10.1038/ng.3973 28991256

[23] Avramopoulos D Recent advances in the genetics of schizophrenia. Mol Neuropsychiatry (2018) 4:35–51. 29998117 10.1159/000488679 PMC6032037

[24] Wood JD Bonath F Kumar S Ross CA Cunliffe VT Disrupted-in-schizophrenia 1 and neuregulin 1 are required for the specification of oligodendrocytes and neurones in the zebrafish brain. Hum Mol Genet (2009) 18:391–404. 18996920 10.1093/hmg/ddn361

[25] Ayhan Y McFarland R Pletnikov MV Animal models of gene-environment interaction in schizophrenia: a dimensional perspective. Prog Neurobiol (2016) 136:1–27. 26510407 10.1016/j.pneurobio.2015.10.002 PMC4707068

[26] Fromer M Roussos P Sieberts SK Johnson JS Kavanagh DH Perumal TM Gene expression elucidates functional impact of polygenic risk for schizophrenia. Nat Neurosci (2016) 19:1442–53. 10.1038/nn.4399 PMC5083142 27668389

[27] Khandaker GM Dantzer R Is there a role for immune-to-brain communication in schizophrenia? Psychopharmacol (Berl) (2016) 233:1559–73. 10.1007/s00213-015-3975-1 PMC4671307 26037944

[28] Nasiadka A Clark MD Zebrafish breeding in the laboratory environment. ILAR J (2012) 53:161–8. 10.1093/ilar.53.2.161 23382347

[29] Wafer LN Jensen VB Whitney JC Gomez TH Flores R Goodwin BS Effects of environmental enrichment on the fertility and fecundity of zebrafish (*Danio rerio*). J Am Assoc Lab Anim Sci (2016) 55:291–4. PMC4865689 27177561

[30] Flurkey K Currer JM Leiter EH Witham B The Jackson laboratory handbook on genetically standardized mice. 6th edition New Mexico: The Jackson Laboratory (2009).

[31] Viana J Wildman N Hannon E Farbos A O’Neill P Moore K Clozapine-induced transcriptional changes in the zebrafish brain. NPJ Schizophr (2020) 6:3. 32015324 10.1038/s41537-019-0092-x PMC6997376

[32] Wienholds E van Eeden F Kosters M Mudde J Plasterk RHA Cuppen E Efficient target-selected mutagenesis in zebrafish. Genome Res (2003) 13:2700–7. 10.1101/gr.1725103 PMC403812 14613981

[33] Moens CB Donn TM Wolf-Saxon ER Ma TP Reverse genetics in zebrafish by TILLING. Brief Funct Genom (2008) 7:454–9. 10.1093/bfgp/eln046 PMC2899843 19028802

[34] Asakawa K Kawakami K Targeted gene expression by the Gal4-UAS system in zebrafish. Dev Growth Differ (2008) 50:391–9. 10.1111/j.1440-169X.2008.01044.x 18482403

[35] Weissman TA Sanes JR Lichtman JW Livet J Generating and imaging multicolor brainbow mice. Cold Spring Harb Protoc (2011) 2011:763–9. 10.1101/pdb.top114 21724826

[36] Thummel R Burket CT Brewer JL Sarras M Li L Perry M Cre-mediated site-specific recombination in zebrafish embryos. Dev Dynam (2005) 233:1366–77. 10.1002/dvdy.20475 15977183

[37] McLellan MA Rosenthal NA Pinto AR Cre-loxP-mediated recombination: general principles and experimental considerations. Curr Protoc Mouse Biol (2017) 7:1–12. 10.1002/cpmo.22 28252198

[38] Hwang WY Fu Y Reyon D Maeder ML Tsai SQ Sander JD Efficient *in vivo* genome editing using RNA-guided nucleases. Nat Biotechnol (2013) 31:227–9. 10.1038/nbt.2501 PMC3686313 23360964

[39] Nemudryi AA Valetdinova KR Medvedev SP Zakian SM TALEN and CRISPR/Cas genome editing systems: tools of discovery. Acta Naturae (2014) 6:19–40. PMC4207558 25349712

[40] Papaleo F Yang F Garcia S Chen J Lu B Crawley JN Dysbindin-1 modulates prefrontal cortical activity and schizophrenia-like behaviors via dopamine/D2 pathways. Mol Psychiatry (2012) 17:85–98. 20956979 10.1038/mp.2010.106 PMC3388848

[41] Papaleo F Burdick MC Callicott JH Weinberger DR Epistatic interaction between COMT and DTNBP1 modulates prefrontal function in mice and in humans. Mol Psychiatry (2014) 19:311–6. 10.1038/mp.2013.133 PMC4845721 24145376

[42] Managò F Mereu M Mastwal S Mastrogiacomo R Scheggia D Emanuele M Genetic disruption of Arc/Arg3.1 in mice causes alterations in dopamine and neurobehavioral phenotypes related to schizophrenia. Cell Rep (2016) 16:2116–28. 10.1016/j.celrep.2016.07.044 PMC5001893 27524619

[43] Cinque S Zoratto F Poleggi A Leo D Cerniglia L Cimino S Behavioral phenotyping of dopamine transporter knockout rats: compulsive traits, motor stereotypies, and anhedonia. Front Psychiatry (2018) 9:43. 29520239 10.3389/fpsyt.2018.00043 PMC5826953

[44] Scheggia D Mastrogiacomo R Mereu M Sannino S Straub RE Armando M Variations in Dysbindin-1 are associated with cognitive response to antipsychotic drug treatment. Nat Commun (2018) 9:2265. 29891954 10.1038/s41467-018-04711-w PMC5995960

[45] Sumitomo A Horike K Hirai K Butcher N Boot E Sakurai T A mouse model of 22q11.2 deletions: molecular and behavioral signatures of Parkinson’s disease and schizophrenia. Sci Adv (2018) 4:eaar6637. 30116778 10.1126/sciadv.aar6637 PMC6093626

[46] Leggio GM Torrisi SA Mastrogiacomo R Mauro D Chisari M Devroye C The epistatic interaction between the dopamine D3 receptor and dysbindin-1 modulates higher-order cognitive functions in mice and humans. Mol Psychiatry (2019). 10.1038/s41380-019-0511-4 31492942

[47] Ettl AK Holzschuh J Driever W The zebrafish mutation m865 affects formation of dopaminergic neurons and neuronal survival, and maps to a genetic interval containing the sepiapterin reductase locus. Anat Embryol (2006) 211:73–86. 17024299 10.1007/s00429-006-0128-7

[48] Boehmler W Carr T Thisse C Thisse B Canfield VA Levenson R D4 dopamine receptor genes of zebrafish and effects of the antipsychotic clozapine on larval swimming behaviour. Genes Brain Behav (2007) 6:155–66. 10.1111/j.1601-183X.2006.00243.x 16764679

[49] Drerup CM Wiora HM Topczewski J Morris JA Disc1 regulates foxd3 and sox10 experssion, affecting neural crest migration and differentiation. Development (2009) 136:2623–32. 10.1242/dev.030577 PMC2709068 19570850

[50] Webb KJ Norton WHJ Trümbach D Meijer AH Ninkovic J Topp S Zebrafish reward mutants reveal novel transcripts mediating the behavioral effects of amphetamine. Genome Biol (2009) 10:R81. 19646228 10.1186/gb-2009-10-7-r81 PMC2728535

[51] Formella I Scott EK Burne THJ Harms LR Liu P Turner KM Transient knockdown of tyrosine hydroxylase during development has persistent effects on behaviour in adult zebrafish (*Danio rerio*). PloS One (2012) 7:e42482. 22879998 10.1371/journal.pone.0042482 PMC3411795

[52] Timme-Laragy AR Karchner SI Hahn ME Gene knockdown by morpholino-modified oligonucleotides in the zebrafish model: applications for developmental toxicology. Methods Mol Biol (2012) 889:51–71. 22669659 10.1007/978-1-61779-867-2_5 PMC4218736

[53] Reissner KJ Sartor GC Vazey EM Dunn TE Aston-Jones G Kalivas PW Use of vivo-morpholinos for control of protein expression in the adult rat brain. J Neurosci Methods (2012) 203:354–60. 10.1016/j.jneumeth.2011.10.009 PMC3857614 22027492

[54] Giacomotto J Carroll AP Rinkwitz S Mowry B Cairns MJ Becker TS Developmental suppression of schizophrenia-associated miR-137 alters sensorimotor function in zebrafish. Transl Psychiatry (2016) 6:e818. 27219344 10.1038/tp.2016.88 PMC5070046

[55] He E Lozano MAG Stringer S Watanabe K Sakamoto K den Oudsten F MIR137 schizophrenia-associated locus controls synaptic function by regulating synaptogenesis, synapse maturation and synaptic transmission. Hum Mol Genet (2018) 27:1879–91. 10.1093/hmg/ddy089 PMC5961183 29635364

[56] Pan YA Freundlich T Weissman TA Schoppik D Wang XC Zimmerman S Zebrabow: multispectral cell labeling for cell tracing and lineage analysis in zebrafish. Development (2013) 140:2835–46. 10.1242/dev.094631 PMC3678346 23757414

[57] Marsden KC Granato M In vivo Ca2+ imaging reveals that decreased dendritic excitability drives startle habituation. Cell Rep (2015) 13:1733–40. 10.1016/j.celrep.2015.10.060 PMC4680997 26655893

[58] Saito A Taniguchi Y Rannals MD Merfeld EB Ballinger MD Koga M Early postnatal GABAa receptor modulation reverses deficits in neuronal maturation in a conditional neurodevelopmental mouse model of DISC1. Mol Psychiatry (2016) 21:1449–59. 10.1038/mp.2015.203 PMC4935661 26728564

[59] Ezran C Karanewsky CJ Pendleton JL Sholtz A Krasnow MR Willick J The mouse lemur, a genetic model organism for primate biology, behavior, and health. Genetics (2017) 206:651–64. 10.1534/genetics.116.199448 PMC5499178 28592502

[60] Sullivan PF Kendler KS Neale MC Schizophrenia as a complex trait. Arch Gen Psychiatry (2003) 60:1187–92. 10.1001/archpsyc.60.12.1187 14662550

[61] Toro CT Deakin JFW Adult neurogenesis and schizophrenia: a window on abnormal early brain development? Schizophr Res (2007) 90:1–14. 10.1016/j.schres.2006.09.030 17123784

[62] van Os J Kenis G Rutten BP The environment and schizophrenia. Nature (2010) 468:203–12. 10.1038/nature09563 21068828

[63] Xu J He G Zhu J Zhou X St Clair D Wang T Prenatal nutritional deficiency reprogrammed postnatal gene expression in mammal brains: implications for schizophrenia. Int J Neuropsychopharmacol (2014) 18:pyu054. 10.1093/ijnp/pyu054 25522397 PMC4360220

[64] McGrath JJ Eyles DW Pedersen CB Anderson C Ko P Burne TH Neonatal vitamin D status and risk of schizophrenia. Arch Gen Psychiatry (2010) 67:889–94. 10.1001/archgenpsychiatry.2010.110 20819982

[65] Selemon LD Zecevic N Schizophrenia: a tale of two critical periods for prefrontal cortical development. Transl Psychiatry (2015) 5:e623. 10.1038/tp.2015.115 26285133 PMC4564568

[66] Flores G Morales-Medina JC Role of the prefrontal cortex in the neonatal ventral hippocampus lesion, an animal model of schizophrenia. J Neurol Neuromed (2016) 1:35–9.

[67] Grayson B Barnes SA Markou A Piercy C Podda G Neill JC Postnatal phencyclidine (PCP) as a neurodevelopmental animal model of schizophrenia pathophysiology and symptomatology: a review. Curr Top Behav Neurosci (2016) 29:403–28. 10.1007/7854_2015_403 26510740

[68] Whitford TJ Rennie CJ Grieve SM Clark CR Gordon E Williams LM Brain maturation in adolescence: concurrent changes in neuroanatomy and neurophysiology. Hum Brain Mapp (2007) 28:228–37. 10.1002/hbm.20273 PMC6871488 16767769

[69] Riccomagno MM Kolodkin AL Sculpting neural circuits by axon and dendrite pruning. Annu Rev Cell Dev Biol (2015) 31:779–805. 26436703 10.1146/annurev-cellbio-100913-013038 PMC4668927

[70] Mondelli V Dazzan P Hepgul N Di Forti M Aas M D’Albenzio A Abnormal cortisol levels during the day and cortisol awakening response in first-episode psychosis: the role of stress and of antipsychotic treatment. Schizophr Res (2010) 116:234–42. 10.1016/j.schres.2009.08.013 PMC3513410 19751968

[71] Carr CP Martins CM Stingel AM Lemgruber VB Juruena MF The role of early life stress in adult psychiatric disorders: a systematic review according to childhood trauma subtypes. J Nerv Ment Dis (2013) 201:1007–20. 10.1097/NMD.0000000000000049 24284634

[72] Geven EJW Klaren PHM The teleost head kidney: integrating thyroid and immune signalling. Dev Comp Immunol (2017) 66:73–83. 27387152 10.1016/j.dci.2016.06.025

[73] Alderman SL Bernier NJ Ontogeny of the corticotropin-releasing factor system in zebrafish. Gen Comp Endocrinol (2009) 164:61–9. 10.1016/j.ygcen.2009.04.007 19366623

[74] Hauser J Feldon J Pryce CR Prenatal dexamethasone exposure, postnatal development, and adulthood prepulse inhibition and latent inhibition in Wistar rats. Behav Brain Res (2006) 175:51–61. 16956676 10.1016/j.bbr.2006.07.026

[75] Marsden CA King MV Fone KCF Influence of social isolation in the rat on serotonergic function and memory - relevance to models of schizophrenia and the role of 5-HT6 receptors. Neuropharmacology (2011) 61:400–7. 10.1016/j.neuropharm.2011.03.003 21414329

[76] Mohn AR Gainetdinov RR Caron MG Koller BH Mice with reduced NMDA receptor expression display behaviors related to schizophrenia. Cell (1999) 98:427–36. 10.1016/s0092-8674(00)81972-8 10481908

[77] Nesan D Vijayan MM Maternal cortisol mediates hypothalamus-pituitary-interrenal axis development in zebrafish. Sci Rep (2016) 6:22582. 10.1038/srep22582 26940285 PMC4778074

[78] Pikulkaew S Benato F Celeghin A Zucal C Skobo T Colombo L The knockdown of maternal glucocorticoid receptor mRNA alters embryo development in zebrafish. Dev Dynam (2011) 240:874–89. 10.1002/dvdy.22586 21360790

[79] Alsop D Vijayan MM Development of the corticosteroid stress axis and receptor expression in zebrafish. American journal of physiology. Am J Physiol Regul Integr Comp Physiol (2008) 1:711–9. 10.1152/ajpregu.00671.2007 18077507

[80] Clark KJ Boczek NJ Ekker SC Stressing zebrafish for behavioral genetics. Rev Neurosci (2011) 22:49–62. 21615261 10.1515/RNS.2011.007 PMC3470424

[81] Idalencio R Kalichak F Santos Rosa JG de Oliveira TA Koakoski G Gusso D Waterborne risperidone decreases stress response in zebrafish. PloS One (2015) 10:e0140800. 10.1371/journal.pone.0140800 26473477 PMC4608780

[82] Grossman L Utterback E Stewart A Gaikwad S Chung KM Suciu C Characterization of behavioral and endocrine effects of LSD on zebrafish. Behav Brain Res (2010) 214:277–84. 10.1016/j.bbr.2010.05.039 20561961

[83] Xu MQ Sun WS Liu BX Feng GY Yu L Yang L Prenatal malnutrition and adult schizophrenia: further evidence from the 1959-1961 chinese famine. Schizophr Bull (2009) 35:568–76. 10.1093/schbul/sbn168 PMC2669578 19155344

[84] Alamy M Bengelloun WA Malnutrition and brain development: an analysis of the effects of inadequate diet during different stages of life in rat. Neurosci Biobehav Rev (2012) 36:1463–80. 10.1016/j.neubiorev.2012.03.009 22487135

[85] Mokler DJ Torres OI Galler JR Morgane PJ Stress-induced changes in extracellular dopamine and serotonin in the medial prefrontal cortex and dorsal hippocampus of prenatally malnourished rats. Brain Res (2007) 1148:226–33. 10.1016/j.brainres.2007.02.031 PMC2706085 17368432

[86] Vucetic Z Totoki K Schoch H Whitaker KW Hill-Smith T Lucki I Early life protein restriction alters dopamine circuitry. Neuroscience (2010) 168:359–70. 10.1016/j.neuroscience.2010.04.010 PMC2873068 20394806

[87] Cruz-Rizzolo RJ Leal LL de Paiva IR Barbosa Ribeiro JO Pimenta T Pinato L Protein maslnutrition during gestation and early life decreases neuronal size in the medial prefrontal cortex of post-pubertal rats. IBRO Rep (2017) 3:65–71. 30135943 10.1016/j.ibror.2017.08.002 PMC6084879

[88] Naderi M Ferrari MCO Chivers DP Niyogi S Maternal exposure to dietary selenium causes dopaminergic hyperfunction and cognitive impairment in zebrafish offspring. Environ Sci Technol (2018) 52:13574–83. 10.1021/acs.est.8b04768 30335985

[89] Kesby JP Cui X Burne TH Eyles DW Altered dopamine ontogeny in the developmentally vitamin D deficient rat and its relevance to schizophrenia. Front Cell Neurosci (2013) 7:111. 10.3389/fncel.2013.00111 23882183 PMC3713405

[90] Kirkbride JB Susser E Kundakovic M Kresovich JK Davey Smith G Relton CL Prenatal nutrition, epigenetics and schizophrenia risk: can we test causal effects? Epigenomics (2012) 4:303–15. 10.2217/epi.12.20 PMC3970193 22690666

[91] Khandaker GM Zimbron J Lewis G Jones PB Prenatal maternal infection, neurodevelopment and adult schizophrenia: a systematic review of population-based studies. Psychol Med (2013) 43:239–57. 10.1017/S0033291712000736 PMC3479084 22717193

[92] Estes ML McAllister KA Maternal immune activation: implications for neuropsychiatric disorders. Science (2016) 353:772–7. 10.1126/science.aag3194 PMC5650490 27540164

[93] Mednick SA Machon RA Huttunen MO Bonett D Adult schizophrenia following prenatal exposure to an influenza epidemic. Arch Gen Psychiatry (1988) 45:189–92. 10.1001/archpsyc.1988.01800260109013 3337616

[94] Brown AS Derkits EJ Prenatal infection and schizophrenia: a review of epidemiologic and translational studies. Am J Psychiatry (2010) 167:261–80. 10.1176/appi.ajp.2009.09030361 PMC3652286 20123911

[95] Sørensen HJ Mortensen EL Reinisch JM Mednick SA Association between prenatal exposure to bacterial infection and risk of schizophrenia. Schizophr Bull (2009) 35:631–7. 10.1093/schbul/sbn121 PMC2669577 18832344

[96] Torrey EF Yolken RH Could schizophrenia be a viral zoonosis transmitted from house cats? Schizophr Bull (1995) 21:167–71. 10.1093/schbul/21.2.167 7631163

[97] Horacek J Flegr J Tintera J Verebova K Spaniel F Novak T Latent toxoplasmosis reduces gray matter density in schizophrenia but not in controls: voxel-based-morphometry (VBM) study. World J Biol Psychiatry (2012) 13:501–9. 10.3109/15622975.2011.573809 21599563

[98] Wischhof L Irrsack E Osorio C Koch M Prenatal LPS-exposure – a neurodevelopmental rat model of schizophrenia – differentially affects cognitive functions, myelination and parvalbumin expression in male and female offspring. Prog Neuropsychopharmacol Biol Psychiatry (2015) 57:17–30. 25455585 10.1016/j.pnpbp.2014.10.004

[99] Kubesova A Tejkalova H Syslova K Kacer P Vondrousova J Tyls F Biochemical, histopathological and morphological profiling of a rat model of early immune stimulation: relation to psychopathology. PloS One (2015) 10:e0115439. 10.1371/journal.pone.0115439 25602957 PMC4300081

[100] Kirsten K Fior D Kreutz LC Barcellos JLG First description of behavior and immune system relationship in fish. Sci Rep (2018) 8:846. 10.1038/s41598-018-19276-3 29339805 PMC5770431

[101] Miyamoto S Miyake N Jarskog LF Fleischhacker WW Lieberman JA Pharmacological treatment of schizophrenia: a critical review of the pharmacology and clinical effects of current and future therapeutic agents. Mol Psychiatry (2012) 17:1206–27. 10.1038/mp.2012.47 22584864

[102] Patel KR Cherian J Gohil K Atkinson D Schizophrenia: overview and treatment options. P T (2014) 39:638–45. PMC4159061 25210417

[103] Melicher T Horacek J Hlinka J Spaniel F Tintera J Ibrahim I White matter changes in first episode psychosis and their relation to the size of sample studied: a DTI study. Schizophr Res (2015) 162:22–8. 10.1016/j.schres.2015.01.029 25660467

[104] Mueller T What is the thalamus in zebrafish? Front Neurosci (2012) 6:1–14. 22586363 10.3389/fnins.2012.00064 PMC3345571

[105] Parker MO Brock AJ Walton RT Brennan CH The role of zebrafish (*Danio rerio*) in dissecting the genetics and neural circuits of executive function. Front Neural Circuits (2013) 7:63. 23580329 10.3389/fncir.2013.00063 PMC3619107

[106] Kozol RA Abrams AJ James DM Buglo E Yan Q Dallman JE Function over form: modeling groups of inherited neurological conditions in zebrafish. Front Mol Neurosci (2016) 9:55. 10.3389/fnmol.2016.00055 27458342 PMC4935692

[107] Gaidica M (2006). labs.gaidi.ca/rat-brain-atlas/ (Accessed April 1, 2020).

[108] Lipska BK Weinberger DR To model a psychiatric disorder in animals: schizophrenia as a reality test. Neuropsychopharmacology (2000) 23:223–39. 10.1016/S0893-133X(00)00137-8 10942847

[109] Daenen EW Wolterink G Van Der Heyden JA Kruse CG Van Ree JM Neonatal lesions in the amygdala or ventral hippocampus disrupt prepulse inhibition of the acoustic startle response; implications for an animal model of neurodevelopmental disorders like schizophrenia. Eur Neuropsychopharm (2003) 13:187–97. 10.1016/s0924-977x(03)00007-5 12729945

[110] Tseng KY Chambers RA Lipska BK The neonatal ventral hippocampal lesion as a heuristic neurodevelopmental model of schizophrenia. Behav Brain Res (2009) 204:295–305. 19100784 10.1016/j.bbr.2008.11.039 PMC2735579

[111] Ganz J Kroehne V Freudenreich D Machate A Geffarth M Braasch I Subdivisions of the adult zebrafish pallium based on molecular marker analysis. F1000Res (2014) 3:308. 10.12688/f1000research.5595.1 25713698 PMC4335597

[112] Vargas JP López JC Portavella M What are the functions of fish brain pallium? Brain Res Bull (2009) 79:436–40. 10.1016/j.brainresbull.2009.05.008 19463910

[113] Cheng RK Jesuthasan SJ Penney TB Zebrafish forebrain and temporal conditioning. Philos Trans R Soc Lond B Biol Sci (2014) 369:20120462. 10.1098/rstb.2012.046 24446496 PMC3895987

[114] Mueller T Wullimann MF An evolutionary interpretation of teleostean forebrain anatomy. Brain Behav Evol (2009) 74:30–42. 19729894 10.1159/000229011

[115] Kimmel CB Warga RM Schilling TF Origin and organization of the zebrafish fate map. Development (1990) 108:581–94. 10.1242/dev.108.4.581 2387237

[116] Nieuwenhuys R The forebrain of actinopterygians revisited. Brain Behav Evol (2009) 73:229–52. 10.1159/000225622 19546532

[117] Nieuwenhuys R The development and general morphology of the telencephalon of actinopterygian fishes: synopsis, documentation and commentary. Brain Struct Funct (2011) 215:141–57. 10.1007/s00429-010-0285-6 PMC3041917 20976604

[118] Yamamoto N Ishikawa Y Yoshimoto M Xue HG Bahaxar N Sawai N A new interpretation on the homology of the teleostean telencephalon based on hodology and a new eversion model. Brain Behav Evol (2007) 69:96–104. 17230017 10.1159/000095198

[119] Franěk M Vaculín S Yamamotová A Šťastný F Bubeníková-Valešová V Rokyta R Pain perception in neurodevelopmental animal models of schizophrenia. Physiol Res (2010) 59:811–9. 10.33549/physiolres.931766 20406041

[120] Aizenberg M Schuman EM Cerebellar-dependent learning in larval zebrafish. J Neurosci (2011) 31:8708–12. 10.1523/JNEUROSCI.6565-10.2011 PMC6622926 21677154

[121] Breier A Su TP Saunders R Carson RE Kolachana BS de Bartolomeis A Schizophrenia is associated with elevated amphetamine-induced synaptic dopamine concentrations: evidence from a novel positron emission tomography method. Proc Natl Acad Sci USA (1997) 94:2569–74. 10.1073/pnas.94.6.2569 PMC20129 9122236

[122] Steeds H Carhart-Harris RL Stone JM Drug models of schizophrenia. Ther Adv Psychopharmacol (2015) 5:43–58. 25653831 10.1177/2045125314557797 PMC4315669

[123] Krajcovic B Fajnerova I Horacek J Kelemen E Kubik S Svoboda J Neural and neuronal discoordination in schizophrenia: from ensembles through networks to symptoms. Acta Physiol (Oxf) (2019) 226:e13282. 10.1111/apha.13282 31002202

[124] Gaskin PL Toledo-Rodriguez M Alexander SP Fone KC Down-regulation of hippocampal genes regulating dopaminergic, GABAergic, and glutamatergic function following combined neonatal phencyclidine and post-weaning social isolation of rats as a neurodevelopmental model for schizophrenia. Int J Neuropsychopharmacol (2016) 19:pyw062. 10.1093/ijnp/pyw062 27382048 PMC5137279

[125] Schwabe K Klein S Koch M Behavioural effects of neonatal lesions of the medial prefrontal cortex and subchronic pubertal treatment with phencyclidine of adult rats. Behav Brain Res (2006) 168:150–60. 10.1016/j.bbr.2005.11.005 16387372

[126] Panula P Sallinen V Sundvik M Kolehmainen J Torkko V Tiittula A Modulatory neurotransmitter systems and behavior: towards zebrafish models of neurodegenerative diseases. Zebrafish (2006) 3:235–47. 10.1089/zeb.2006.3.235 18248264

[127] Seibt KJ Piato AL da Luz Oliveira R Capiotti KM Vianna MR Bonan CD Antipsychotic drugs reverse MK-801-induced cognitive and social interaction deficits in zebrafish (*Danio rerio*). Behav Brain Res (2011) 224:135–9. 10.1016/j.bbr.2011.05.034 21669233

[128] Rico EP de Oliveira DL Rosemberg DB Mussulini BH Bonan CD Dias RD Expression and functional analysis of Na+-dependent glutamate transporters from zebrafish brain. Brain Res Bull (2010) 81:517–23. 10.1016/j.brainresbull.2009.11.011 19941938

[129] Rico EP Rosemberg DB Seibt KJ Capiotti KM Da Silva RS Bonan CD Zebrafish neurotransmitter systems as potential pharmacological and toxicological targets. Neurotoxicol Teratol (2011) 33:608–17. 10.1016/j.ntt.2011.07.007 21907791

[130] Rubio MD Drummond JB Meador-Woodruff JH Glutamate receptor abnormalities in schizophrenia: implications for innovative treatments. Biomol Ther (2012) 20:1–18. 10.4062/biomolther.2012.20.1.001 PMC3792192 24116269

[131] Adell A Jiménez-Sánchez L López-Gill X Romón T Is the acute NMDA receptor hypofunction a valid model of schizophrenia? Schizophr Bull (2012) 38:9–14. 21965469 10.1093/schbul/sbr133 PMC3245580

[132] Kim SY Lee H Kim HJ Bang E Lee SH Lee DW *In vivo* and *ex vivo* evidence for ketamine-induced hyperglutamatergic activity in the cerebral cortex of the rat: potential relevance to schizophrenia. NMR BioMed (2011) 24:1235–42. 10.1002/nbm.1681 21560175

[133] Balla A Nattini ME Sershen H Lajtha A Dunlop DS Javitt DC GABAB/NMDA receptor interaction in the regulation of extracellular dopamine levels in rodent prefrontal cortex and striatum. Neuropharmacology (2009) 56:915–21. 10.1016/j.neuropharm.2009.01.021 PMC4681299 19371582

[134] Laruelle M Schizophrenia: from dopaminergic to glutamatergic interventions. Curr Opin Pharmacol (2014) 14:97–102. 24524997 10.1016/j.coph.2014.01.001

[135] Laurelle M Kegeles L Abi-Dargham A Glutamate, dopamine, and schizophrenia from pathology to treatment. Ann N Y Acad Sci (2003) 1003:138–58. 10.1196/annals.1300.063 14684442

[136] Quednow BB Geyer MA Halberstadt AL Serotonin and schizophrenia. Handb Behav Neurosci (2010) 21:585–620.

[137] Stahl SM Beyond the dopamine hypothesis of schizophrenia to three neural networks of psychosis: dopamine, serotonin, and glutamate. CNS Spectr (2018) 23:187–91. 10.1017/S1092852918001013 29954475

[138] Brunelin J Fecteau S Suaud-Chagny MF Abnormal striatal dopamine transmission in schizophrenia. Curr Med Chem (2013) 20:397–404. 23157632 10.2174/0929867311320030011 PMC3866953

[139] Gruber O Chadha Santuccione A Aach H Magnetic resonance imaging in studying schizophrenia, negative symptoms, and the glutamate system. Front Psychiatry (2014) 5:32. 24765078 10.3389/fpsyt.2014.00032 PMC3982059

[140] Haleem DJ 5-HT1A receptor-dependent control of nigrostriatal dopamine neurotransmission in the pharmacotherapy of Parkinson’s disease and schizophrenia. Behav Pharmacol (2015) 26:45–58. 25503261 10.1097/FBP.0000000000000123

[141] Rink E Wullimann MF The teleostean (zebrafish) dopaminergic system ascending to the subpallium (striatum) is located in the basal diencephalon (posterior tuberculum). Brain Res (2001) 889:316–30. 10.1016/s0006-8993(00)03174-7 11166725

[142] Rink E Wullimann MF Connections of the ventral telencephalon and tyrosine hydroxylase distribution in the zebrafish brain (*Danio rerio*) lead to identification of an ascending dopaminergic system in a teleost. Brain Res Bull (2002) 57:385–7. 10.1016/s0361-9230(01)00696-7 11922994

[143] Guo S Wilson SW Cooke S Chitnis AB Driever W Rosenthal A Mutations in the zebrafish unmask shared regulatory pathways controlling the development of catecholaminergic neurons. Dev Biol (1999) 208:473–87. 10.1006/dbio.1999.9204 10191060

[144] Schweizer J Lohr H Filippi A Driever W Dopaminergic and noradrenergic circuit development in zebrafish. Dev Neurobiol (2012) 72:256–68. 10.1002/dneu.20911 21567980

[145] Maximino C Herculano AM A review of monoaminergic neuropsychopharmacology in zebrafish. Zebrafish (2010) 7:359–78. 10.1089/zeb.2010.0669 21158565

[146] Tarazi FI Neuropharmacology of dopamine receptors: implications in neuropsychiatric diseases. J Sci Res Med Sci (2001) 3:93–104. 24019715 PMC3174705

[147] Seeman P Dopamine D2 receptors as treatment targets in schizophrenia. Clin Schizophr Relat Psychoses (2010) 4:56–73. 20643630

[148] Boehmler W Obrecht-Pflumio S Canfield V Thisse C Thisse B Levenson R Evolution and expression of D2 and D3 dopamine receptor genes in zebrafish. Dev Dynam (2004) 230:481–93. 10.1002/dvdy.20075 15188433

[149] Ek F Malo M Åberg Andersson M Wedding C Kronborg J Svensson P Behavioral analysis of dopaminergic activation in zebrafish and rats reveals similar phenotypes. ACS Chem Neurosci (2016) 7:633–46. 10.1021/acschemneuro.6b00014 26947759

[150] Panula P Chen YC Priyadarshini M Kudo H Semenova S Sundvik M The comparative neuroanatomy and neurochemistry of zebrafish CNS systems of relevance to human neuropsychiatric diseases. Neurobiol Dis (2010) 40:46–57. 20472064 10.1016/j.nbd.2010.05.010

[151] Mueller T Dong Z Berberoglu MA Guo S The dorsal pallium in zebrafish, *Danio rerio* (Cyprinidae, Teleostei). Brain Res (2011) 1381:95–105. 21219890 10.1016/j.brainres.2010.12.089 PMC3052766

[152] Halberstadt AL Geyer MA Serotonergic hallucinogens as translational models relevant to schizophrenia. Int J Neuropsychopharmacol (2013) 16:2165–80. 10.1017/S1461145713000722 PMC3928979 23942028

[153] Hollister LE Drug-induced psychoses and schizophrenic reactions: a critical comparison. Ann NY Acad Sci (1962) 96:80–92. 13908450 10.1111/j.1749-6632.1962.tb50103.x

[154] Muguruza C Moreno JL Umali A Callado LF Meana JJ González-Maeso J Dysregulated 5-HT2A receptor binding in postmortem frontal cortex of schizophrenic subject. Eur Neuropsychopharm (2013) 23:852–64. 10.1016/j.euroneuro.2012.10.006 PMC3586752 23176747

[155] Fink KB Göthert M 5-HT receptor regulation of neurotransmitter release. Pharmacol Rev (2007) 59:360–417. 18160701 10.1124/pr.107.07103

[156] UniProt (2002). Available at: www.uniprot.org/uniprot/?query=5-ht&sort=score (Accessed March 3, 2020).

[157] Lillesaar C Stigloher C Tannhäuser B Wullimann MF Bally-Cuif L Axonal projections originating from raphe serotonergic neurons in the developing and adult zebrafish, *Danio rerio*, using transgenics to visualize raphe-specific pet1 expression. J Comp Neurol (2009) 512:158–82. 10.1002/cne.21887 19003874

[158] Herculano AM Maximino C Serotonergic modulation of zebrafish behavior: towards a paradox. Prog Neuropsychopharmacol Biol Psychiatry (2014) 55:50–66. 24681196 10.1016/j.pnpbp.2014.03.008

[159] Robinson KSL Stewart A Cachat J Landsman S Gebhardt M Kalueff AV Psychopharmacological effects of acute exposure to kynurenic acid (KYNA) in zebrafish. Pharmacol Biochem Behav (2013) 108:54–60. 23583441 10.1016/j.pbb.2013.04.002

[160] Stewart AM Ullmann JFP Norton WH Parker MO Brennan CH Gerlai R Molecular psychiatry of zebrafish. Mol Psychiatry (2015) 20:2–17. 25349164 10.1038/mp.2014.128 PMC4318706

[161] Oltrabella F Melgoza A Nguyen B Guo S Role of the endocannabinoid system in vertebrates: emphasis on the zebrafish model. Dev Growth Differ (2017) 59:194–210. 28516445 10.1111/dgd.12351 PMC5636690

[162] Williams FE Messer W Muscarinic acetylcholine receptors in the brain of the zebrafish (*Danio rerio*) measured by radioligand binding techniques. Comp Biochem Physiol C Toxicol Pharmacol (2004) 137:349–53. 10.1016/j.cca.2004.03.002 15228953

[163] Akhtar MT Ali S Rashidi H van der Kooy F Verpoorte R Richardson MK Developmental effects of cannabinoids on zebrafish larvae. Zebrafish (2013) 10:283–93. 10.1089/zeb.2012.0785 23789728

[164] Menezes FP Bonan C Evaluation of age-dependent response to NMDA receptor antagonism in zebrafish. Zebrafish (2015) 12:137–43. 10.1089/zeb.2014.1018 25602300

[165] Mahabir S Chatterjee D Buske C Gerlai R Maturation of shoaling in two zebrafish strains: a behavioral and neurochemical analysis. Behav Brain Res (2013) 247:1–8. 23518435 10.1016/j.bbr.2013.03.013 PMC3646909

[166] Ralph RJ Caine SB Dopamine D1 and D2 agonist effects on prepulse inhibition and locomotion: comparison of Sprague-Dawley rats to Swiss-Webster, 129X1/SvJ, C57BL/6J, and DBA/2J mice. J Pharmacol Exp Ther (2005) 312:733–41. 10.1124/jpet.104.074468 15494551

[167] Swain HA Sigstad C Scalzo FM Effects of dizocilpine (MK-801) on circling behavior, swimming activity, and place preference in zebrafish (*Danio rerio*). Neurotoxicol Teratol (2004) 26:725–9. 10.1016/j.ntt.2004.06.009 15451036

[168] Norton W Bally-Cuif L Adult zebrafish as a model organism for behavioural genetics. BMC Neurosci (2010) 11:90. 10.1186/1471-2202-11-90 20678210 PMC2919542

[169] Kalueff AV Stewart AM Gerlai R Zebrafish as an emerging model for studying complex brain disorders. Trends Pharmacol Sci (2014) 35:63–75. 24412421 10.1016/j.tips.2013.12.002 PMC3913794

[170] Fontana BD Mezzomo NJ Kalueff AV Rosemberg DB The developing utility of zebrafish models of neurological and neuropsychiatric disorders: a critical review. Exp Neurol (2018) 299:157–71. 10.1016/j.expneurol.2017.10.004 28987462

[171] Sturgeon DR Fessler RG Meltzer HY Behavioral rating scales for assessing phencyclidine-induced locomotor activity, stereotypes behavior and ataxia in rats. Eur J Pharmacol (1979) 59:169–79. 10.1016/0014-2999(79)90279-6 575093

[172] Roberts AC Reichl J Song MY Dearinger AD Moridzadeh N Lu ED Habituation of the C-Start response in larval zebrafish exhibits several distinct phases and sensitivity to NMDA receptor blockade. PloS One (2011) 6:e29132. 10.1371/journal.pone.0029132 22216183 PMC3247236

[173] Scheggi S De Montis MG Gambarana C Making sense of rodent models of anhedonia. Int J Neuropsychopharmacol (2018) 21:1049–65. 10.1093/ijnp/pyy083 PMC6209858 30239762

[174] Mansbach RS Geyer MA Braff DL Dopaminergic stimulation disrupts sensorimotor gating in the rat. Psychopharmacol (Berl) (1988) 94:507–14. 10.1007/BF00212846 3131796

[175] Ericson EL Ahlenius S Phencyclidine-induced disruption of an aversely motivated two-choice successive discrimination in the rat. Psychopharmacology (1990) 102:171–4. 10.1007/BF02245918 1980371

[176] Butelman ER The effect of NMDA antagonists in the radial arm maze task with an interposed delay. Pharmacol Biochem Behav (1990) 35:533–6. 10.1016/0091-3057(90)90285-p 2160086

[177] Barnett SA The rat: a study in behavior. Chicago: The University of Chicago Press (1976).

[178] Kalueff AV Illustrated zebrafish neurobehavioral glossary. In: Kalueff, AV, editor: The Rights and Wrongs of Zebrafish: Behavioral Phenotyping of Zebrafish. Cham, CH: Springer International Publishing (2017). p. 291–317.

[179] Spence R Gerlach G Lawrence C Smith C The behaviour and ecology of the zebrafish, *Danio rerio*. Biol Rev (2008) 83:13–34. 18093234 10.1111/j.1469-185X.2007.00030.x

[180] Irons TD Kelly PE Hunter DL Macphail RC Padilla S Acute administration of dopaminergic drugs has differential effects on locomotion in larval zebrafish. Pharmacol. Pharmacol Biochem Behav (2013) 103:792–813. 23274813 10.1016/j.pbb.2012.12.010 PMC3640837

[181] Hunt MJ Raynaud B Garcia R Ketamine dose-dependently induces high-frequency oscillations in the nucleus accumbens in freely moving rats. Biol Psychiatry (2006) 60:1206–14. 10.1016/j.biopsych.2006.01.020 16650831

[182] Powell CM Miyakawa T Schizophrenia-relevant behavioral testing in rodent models: a uniquely human disorder? Biol Psychiatry (2006) 59:1198–207. 10.1016/j.biopsych.2006.05.008 PMC3928106 16797265

[183] Geyer MA Ellenbroek B Animal behavior models of the mechanisms underlying antipsychotic atypicality. Prog Neuropsychopharmacol Biol Psychiatry (2003) 27:1071–9. 10.1016/j.pnpbp.2003.09.003 14642967

[184] Ennaceur A Michalikova S Chazot PL Models of anxiety: responses of rats to novelty in an open space and an enclosed space. Behav Brain Res (2006) 171:26–49. 16678277 10.1016/j.bbr.2006.03.016

[185] Ohl F Arndt SS van der Staay FJ Pathological anxiety in animals. Vet J (2008) 175:18–26. 17321766 10.1016/j.tvjl.2006.12.013

[186] Egan RJ Bergner CL Hart PC Cachat JM Canavello PR Elegante MF Understanding behavioral and physiological phenotypes of stress and anxiety in zebrafish. Behav Brain Res (2009) 205:38–44. 19540270 10.1016/j.bbr.2009.06.022 PMC2922906

[187] Páleníček T Fujáková M Brunovský M Balíková M Horáček J Gorman I Electroencephalographic spectral and coherence analysis of ketamine in rats: correlation with behavioral effects and pharmacokinetics. Neuropsychobiology (2011) 63:202–18. 10.1159/000321803 21422767

[188] Kimmel CB Ballard WW Kimmel SR Ullmann B Schilling TF Stages of embryonic development of the zebrafish. Dev Dynam (1995) 203:253–310. 10.1002/aja.1002030302 8589427

[189] Creese I Iversen SD The role of forebrain dopamine systems in amphetamine induced stereotyped behavior in the rat. Psychopharmacologia (1974) 39:345–57. 10.1007/BF00422974 4615333

[190] Burket JA Cannon WR Jacome LF Deutsch SI MK-801, a noncompetitive NMDA receptor antagonist, elicits circling behavior in the genetically inbred Balb/c mouse strain. Brain Res Bull (2010) 83:337–9. 10.1016/j.brainresbull.2010.08.014 20813169

[191] Bracha HS Asymmetric rotational (circling) behavior, a dopamine-related asymmetry: preliminary findings in unmedicated and never-medicated schizophrenic patients. Biol Psychiatry (1987) 22:995–1003. 3607140 10.1016/0006-3223(87)90009-6

[192] Riehl R Kyzar E Allain A Green J Hook M Monnig L Behavioral and physiological effects of acute ketamine exposure in adult zebrafish. Neurotoxicol Teratol (2011) 33:658–67. 10.1016/j.ntt.2011.05.011 21683787

[193] Zakhary SM Ayubcha D Ansari F Kamran K Karim M Leheste JR A behavioral and molecular analysis of ketamine in zebrafish. Synapse (2011) 65:160–7. 10.1002/syn.20830 PMC2978795 20623473

[194] Jansen LM Gispen de Wied CC Kahn RS Selective impairments in the stress response in schizophrenic patients. Psychopharmacol (Berl) (2000) 149:319–25. 10.1007/s002130000381 10823414

[195] Lange C Deutschenbaur L Borgwardt S Lang UE Walter M Huber CG Experimentally induced psychosocial stress in schizophrenia spectrum disorders: a systematic review. Schizophr Res (2017) 182:4–12. 27733301 10.1016/j.schres.2016.10.008

[196] Budick SA O’Malley DM Locomotor repertoire of the larval zebrafish: swimming, turning and prey capture. J Exp Biol (2000) 203:2565–79. 10.1242/jeb.203.17.2565 10934000

[197] Cachat J Stewart A Grossman L Gaikwad S Kadri F Chung KM Measuring behavioral and endocrine responses to novelty stress in adult zebrafish. Nat Protoc (2010) 5:1786–99. 10.1038/nprot.2010.140 21030954

[198] Griffiths BB Schoonheim PJ Ziv L Voelker L Baier H Gahtan E A zebrafish model of glucocorticoid resistance shows serotonergic modulation of the stress response. Front Behav Neurosci (2012) 6:68. 10.3389/fnbeh.2012.00068 23087630 PMC3468897

[199] Karnik I Gerlai R Can zebrafish learn spatial tasks? An empirical analysis of place single CS-US associative learning. Behav Brain Res (2012) 233:415–21. 10.1016/j.bbr.2012.05.024 PMC3402691 22633962

[200] Bubeníková V Votava M Horáček J Páleníček T Dockery C The effect of zotepine, risperidone, clozapine and olanzapine on MK-801-disrupted sensorimotor gating. Pharmacol Biochem Behav (2005) 80:591–6. 10.1016/j.pbb.2005.01.012 15820528

[201] Suriyampola PS Shelton DS Shukla R Roy T Bhat A Martins EP Zebrafish social behavior in the wild. Zebrafish (2016) 13:1–8. 10.1089/zeb.2015.1159 26671510

[202] Spence R Fatema MK Reichard M Huq KA Wahab MA Ahmed ZF The distribution and habitat preferences of the zebrafish in Bangladesh. J Fish Biol (2006) 69:1435–48.

[203] Koolhaas JM Schuurman T Wiepkema PR The organization of intraspecific agonistic behaviour in the rat. Prog Neurobiol (1980) 15:247–68. 10.1016/0301-0082(80)90024-6 7005965

[204] Rung JP Carlsson A Rydén Markinhuhta K Carlsson ML (+)-MK-801 induced social withdrawal in rats; a model for negative symptoms of schizophrenia. Prog Neuropsychopharmacol Biol Psychiatry (2005) 29:827–32. 10.1016/j.pnpbp.2005.03.004 15916843

[205] McGraw LA Young LJ The prairie vole: an emerging model organism for understanding the social brain. Trends Neurosci (2010) 33:103–9. 10.1016/j.tins.2009.11.006 PMC2822034 20005580

[206] Pike TW Samanta M Lindström J Royle NJ Behavioural phenotype affects social interactions in an animal network. Proc R Soc B (2008) 275:2515–20. 10.1098/rspb.2008.0744 PMC2603202 18647713

[207] Bell AM Sih A Exposure to predation generates personality in threespined sticklebacks (*Gasterosteus aculeatus*). Ecol Lett (2007) 10:828–34. 10.1111/j.1461-0248.2007.01081.x 17663716

[208] Horká P Horký P Randák T Turek J Rylková K Slavík O Radio-telemetry shows differences in the behaviour of wild and hatchery-reared European grayling Thymallus thymallus in response to environmental variables. J Fish Biol (2015) 86:544–57. 10.1111/jfb.12575 25604702

[209] Horká P Sychrová O Horký P Slavík O Švátora M Petrusek A Feeding habits of the alien brook trout Salvelinus fontinalis and the native brown trout Salmo trutta in Czech mountain streams. Knowl Manag Aquat Ec (2017) 418:6. 10.1051/kmae/2016038

[210] Slavík O Horký P Maciak M Horká P Langrová I Diel movement of brown trout, Salmo trutta, is reduced in dense populations with high site fidelity. Ecol Evol (2018) 8:4495–507. 10.1002/ece3.3981 PMC5938464 29760890

[211] Horan WP Kring AM Blanchard JJ Anhedonia in schizophrenia: a review of assessment strategies. Schizophr Bull (2006) 32:259–73. 10.1093/schbul/sbj009 PMC2632206 16221997

[212] Gard DE Kring AM Gard GM Horan WP Green MF Anhedonia in schizophrenia: distinctions between anticipatory and consummatory pleasure. Schizophr Res (2007) 93:253–60. 10.1016/j.schres.2007.03.008 PMC1986826 17490858

[213] Strauss GP The emotion paradox of anhedonia in schizophrenia: or is it? Schizophr Bull (2013) 39:247–50. 10.1093/schbul/sbs192 PMC3576151 23328158

[214] Moreau JL Simulating the anhedonia symptom of depression in animals. Dialogues Clin Neurosci (2002) 4:351–60. 10.31887/DCNS.2002.4.4/jlmoreau PMC3181703 22034464

[215] Nguyen M Stewart AM Kalueff AV Aquatic blues: modeling depression and antidepressant action in zebrafish. Prog Neuropsychopharmacol Biol Psychiatry (2014) 55:26–39. 24657522 10.1016/j.pnpbp.2014.03.003

[216] Simpson EH Waltz JA Kellendonk C Balsam PD Schizophrenia in translation: dissecting motivation in schizophrenia and rodents. Schizophr Bull (2012) 38:1111–7. 10.1093/schbul/sbs114 PMC3494038 23015686

[217] Wilson CA Koenig JI Social interaction and social withdrawal in rodents as readouts for investigating the negative symptoms of schizophrenia. Eur Neuropsychopharm (2014) 24:759–73. 10.1016/j.euroneuro.2013.11.008 PMC4481734 24342774

[218] Scerbina T Chatterjee D Gerlai R Dopamine receptor antagonism disrupts social preference in zebrafish, a strain comparison study. Amino Acids (2012) 43:2059–72. 10.1007/s00726-012-1284-0 PMC3425734 22491827

[219] Clapcote SJ Lipina TV Millar JK Mackie S Christie S Ogawa F Behavioral phenotypes of Disc1 missense mutations in mice. Neuron (2007) 54:387–402. 17481393 10.1016/j.neuron.2007.04.015

[220] Harvey PD Strassing M Predicting the severity of everyday functional disability in people with schizophrenia: cognitive deficits, functional capacity, symptoms, and health status. World Psychiatry (2012) 11:73–9. 10.1016/j.wpsyc.2012.05.004 PMC3363376 22654932

[221] Judd LL McAdams L Budnick B Braff DL Sensory gating deficits in schizophrenia: new results. Am J Psychiatry (1992) 149:488–93. 10.1176/ajp.149.4.488 1554034

[222] Kupferschmidt DA Gordon JA The dynamics of disordered dialogue: prefrontal, hippocampal and thalamic miscommunication underlying working memory deficits in schizophrenia. BNA (2018) 2:1–15. 10.1177/2398212818771821 PMC6497416 31058245

[223] Carter JD Bizzell J Kim C Bellion C Carpenter KL Dichter G Attention deficits in schizophrenia - preliminary evidence of dissociable transient and sustained deficits. Schizophr Res (2010) 122:104–12. 10.1016/j.schres.2010.03.019 PMC2933272 20554160

[224] Williams LE Blackford JU Luksik A Gauthier I Heckers S Reduced habituation in patients with schizophrenia. Schizophr Res (2013) 151:124–32. 10.1016/j.schres.2013.10.017 PMC3908315 24200419

[225] Wadehra S Pruitt P Murphy ER Diwadkar VA Network dysfunction during associative learning in schizophrenia: increased activation, but decreased connectivity: an fMRI study. Schizophr Res (2013) 148:38–49. 23759649 10.1016/j.schres.2013.05.010

[226] Hammer TB Oranje B Fagerlund B Bro H Glenthøj BY Stability of prepulse inhibition and habituation of the startle reflex in schizophrenia: a 6-year follow-up study of initially antipsychotic-naive, first-episode schizophrenia patients. Int J Neuropsychopharmacol (2011) 14:913–25. 10.1017/S1461145711000034 21294942

[227] Swerdlow NR Braff DL Geyer MA Sensorimotor gating of the startle reflex: what we said 25 years ago, what has happened since then, and what comes next. J Psychopharmacol (Oxford) (2016) 30:1072–81. 10.1177/0269881116661075 PMC6036900 27539931

[228] Burgess HA Granato M Sensorimotor gating in larval zebrafish. J Neurosci (2007) 27:4984–94. 10.1523/JNEUROSCI.0615-07.2007 PMC6672105 17475807

[229] Bhandiwad AA Zeddies DG Raible DW Rubel EW Sisneros JA Auditory sensitivity of larval zebrafish (*Danio rerio*) measured using a behavioral prepulse inhibition assay. J Exp Biol (2013) 216:3504–13. 10.1242/jeb.087635 PMC3749908 23966590

[230] Rohleder C Wiedermann D Neumaier B Drzezga A Timmermann L Graf R The functional networks of prepulse inhibition: neuronal connectivity analysis based on FDG-PET in awake and unrestrained rats. Front Behav Neurosci (2016) 10:148. 10.3389/fnbeh.2016.00148 27493627 PMC4954847

[231] Swerdlow NR Light GA Sensorimotor gating deficits in schizophrenia: advancing our understanding of the phenotype, its neural circuitry and genetic substrate. Schizophr Res (2018) 198:1–5. 29525460 10.1016/j.schres.2018.02.042 PMC6103885

[232] Eaton RC DiDomenico R Nissanov J Role of the Mauthner cell in sensorimotor integration by the brain stem escape network. Brain Behav Evol (1991) 37:272–85. 10.1159/000114365 1933251

[233] Best JD Berghmans S Hunt JJ Clarke SC Fleming A Goldsmith P Non-associative learning in larval zebrafish. Neuropsychopharmacology (2008) 33:1206–15. 10.1038/sj.npp.1301489 17581529

[234] Valsamis B Schmid S Habituation and prepulse inhibition of acoustic startle in rodents. J Vis Exp (2011) 55:e3446. 10.3791/3446 PMC3217252 21912367

[235] Roberts AC Bill BR Glanzman DL Learning and memory in zebrafish larvae. Front Neural Circuit (2013) 7:126. 10.3389/fncir.2013.00126 PMC3731533 23935566

[236] Wolman MA Jain RA Liss L Granato M Chemical modulation of memory formation in larval zebrafish. Proc Natl Acad Sci USA (2011) 108:15468–73. 10.1073/pnas.1107156108 PMC3174630 21876167

[237] Diwadkar VA Flaugher B Jones T Zalányi L Ujfalussy B Keshavan MS Impaired associative learning in schizophrenia: behavioral and computational studies. Cognit Neurodyn (2008) 2:207–19. 10.1007/s11571-008-9054-0 PMC2518754 19003486

[238] Harloe JP Thorpe AJ Lichtman AH Differential endocannabinoid regulation of extinction in appetitive and aversive Barnes maze tasks. Learn Mem (2008) 15:806–9. 10.1101/lm.1113008 PMC2632809 18957525

[239] Sison M Gerlai R Associative learning performance is impaired in zebrafish (*Danio rerio*) by the NMDA-R antagonist MK-801. Neurobiol Learn Mem (2011) 96:230–7. 10.1016/j.nlm.2011.04.016 PMC3148332 21596149

[240] de P Cognato G Bortolotto JW Blazina AR Christoff RR Lara DR Vianna MR Y-maze memory task in zebrafish (*Danio rerio*): the role of glutamatergic and cholinergic systems on the acquisition and consolidation periods. Neurobiol Learn Mem (2012) 98:321–8. 10.1016/j.nlm.2012.09.008 23044456

[241] Xu X Scott-Scheiern T Kempker L Simons K Active avoidance conditioning in zebrafish (*Danio rerio*). Neurobiol Learn Mem (2007) 87:72–7. 10.1016/j.nlm.2006.06.002 16861014

[242] Pirkola T Tuulio-Henriksson A Glahn D Kieseppä T Haukka J Kaprio J Spatial working memory function in twins with schizophrenia and bipolar disorder. Biol Psychiatry (2005) 58:930–6. 10.1016/j.biopsych.2005.05.041 16112657

[243] Orellana G Slachevsky A Executive functioning in schizophrenia. Front Psychiatry (2013) 4:35. 23805107 10.3389/fpsyt.2013.00035 PMC3690455

[244] Goetghebeur P Dias R Comparison of haloperidol, risperidone, sertindole, and modafinil to reverse an attentional set-shifting impairment following subchronic PCP administration in the rat - a back translational study. Psychopharmacol (Berl) (2009) 202:287–93. 10.1007/s00213-008-1132-9 18392753

[245] Young JW Geyer MA Rissling AJ Sharp RF Eyler LT Asgaard GL Reverse translation of the rodent 5C-CPT reveals that the impaired attention of people with schizophrenia is similar to scopolamine-induced deficits in mice. Transl Psychiatry (2013) 3:e324. 10.1038/tp.2013.82 24217494 PMC3849961

[246] Esnal A Sánchez-González A Río-Álamos C Oliveras I Cañete T Blázquez G Prepulse inhibition and latent inhibition deficits in Roman high-avoidance vs. Roman low-avoidance rats: modeling schizophrenia-related features. Physiol Behav (2016) 163:267–73. 10.1016/j.physbeh.2016.05.020 27184235

[247] Barnes SA Young JW Markou A Adham N Gyertyán I Kiss B The effects of cariprazine and aripiprazole on PCP-induced deficits on attention assessed in the 5-choice serial reaction time task. Psychopharmacol (Berl) (2018) 235:1403–14. 10.1007/s00213-018-4857-0 PMC5920008 29473089

[248] Stark KL Xu B Bagchi A Lai WS Liu H Hsu R Altered brain microRNA biogenesis contributes to phenotypic deficits in a 22q11-deletion mouse model. Nat Genet (2008) 40:751–60. 10.1038/ng.138 18469815

[249] Bardgett ME Baum KT O’Connell SM Lee NM Hon JC Effects of risperidone on locomotor activity and spatial memory in rats with hippocampal damage. Neuropharmacology (2006) 51:1156–62. 10.1016/j.neuropharm.2006.07.014 16934300

[250] Wang AL Chao OY Yang YM Trossbach SV Müller CP Korth C Anxiogenic-like behavior and deficient attention/working memory in rats expressing the human DISC1 gene. Pharmacol Biochem Behav (2019) 179:73–9. 10.1016/j.pbb.2019.02.005 30779934

[251] Monte AS Mello BSF Borella VCM da Silva Araujo T da Silva FER de Sousa FCF Two-hit model of schizophrenia induced by neonatal immune activation and peripubertal stress in rats: study of sex differences and brain oxidative alterations. Behav Brain Res (2017) 331:30–7. 10.1016/j.bbr.2017.04.057 28527693

[252] Murray BG Davies DA Molder JJ Howland JG Maternal immune activation during pregnancy in rats impairs working memory capacity of the offspring. Neurobiol Learn Mem (2017) 141:150–6. 10.1016/j.nlm.2017.04.005 28434949

[253] de Castro MR Lima JV de Freitas DP de Souza Valente R Dummer NS de Aguiar RB Behavioral and neurotoxic effects of arsenic exposure in zebrafish (*Danio rerio*, Teleostei: Cyprinidae). Comp Biochem Physiol C Toxicol Pharmacol (2009) 150:337–42. 10.1016/j.cbpc.2009.05.017 19501674

[254] Bahl A Engert F Neural circuits for evidence accumulation and decision making in larval zebrafish. Nat Neurosci (2020) 23:94–102. 31792464 10.1038/s41593-019-0534-9 PMC7295007

[255] Mailman RB Murthy V Third generation atipsychotic drugs: partial agonism or receptor functional selectivity? Curr Pharm Des (2010) 16:488–501. 19909227 10.2174/138161210790361461 PMC2958217

[256] Aringhieri S Carli M Kolachalam S Verdesca V Cini E Rossi M Molecular targets of atypical antipsychotics: from mechanism of action to clinical differences. Pharmacol Ther (2018) 192:20–41. 29953902 10.1016/j.pharmthera.2018.06.012

[257] Lipska BK Weinberger DR Subchronic treatment with haloperidol and clozapine in rats with neonatal excitotoxic hippocampal damage. Neuropsychopharmacology (1994) 10:199–205. 7916917 10.1038/npp.1994.22

[258] McOmish CE Burrows E Howard M Scarr E Kim D Shin HS Phospholipase C-beta1 knockout mice exhibit endophenotypes modeling schizophrenia which are rescued by environmental enrichment and clozapine administration. Mol Psychiatry (2008) 13:661–72. 10.1038/sj.mp.4002046 17667964

[259] Pezze MA Dalley JW Robbins TW Remediation of attentional dysfunction in rats with lesions of the medial prefrontal cortex by intra-accumbens administration of the dopamine D2/3 receptor antagonist sulpiride. Psychopharmacol (Berl) (2009) 202:307–13. 10.1007/s00213-008-1384-4 18985321

[260] Seibt KJ da Luz Oliveira R Zimmermann FF Capiotti KM Boggo MR Ghisleni G Antipsychotic drugs prevent the motor hyperactivity induced by psychotomimetic MK-801 in zebrafish (*Danio rerio*). Behav Brain Res (2010) 214:417–22. 10.1016/j.bbr.2010.06.014 20600350

[261] Qiao H Noda Y Kamei H Nagai T Furukawa H Miura H Clozapine, but not haloperidol, reverses social behavior deficit in mice during withdrawal from chronic phencyclidine treatment. Neuroreport (2001) 12:11–5. 10.1097/00001756-200101220-00010 11201068

[262] Bruins Slot LA Kleven MS Newman-Tancredi A Effects of novel antipsychotics with mixed D2 antagonist/5-HT 1A agonist properties on PCP-induced social interaction deficits in the rat. Neuropharmacology (2005) 49:996–1006. 16009387 10.1016/j.neuropharm.2005.05.013

[263] Chartoff EH Heusner CL Palmiter RD Dopamine is not required for the hyperlocomotor response to NMDA receptor antagonists. Neuropsychopharmacology (2005) 30:1324–33. 10.1038/sj.npp.1300678 15688082

[264] Sams-Dodd F Effect of novel antipsychotic drugs on phencyclidine-induced stereotyped behaviour and social isolation in the rat social interaction test. Behav Pharmacol (1997) 8:196–215. 9833015

[265] Kohnomi S Suemaru K Kawasaki H Araki H Effect of aripiprazole on 5-HT2 receptor-mediated wet-dog shake responses and disruption of prepulse inhibition in rats. J Pharmacol Sci (2008) 106:645–50. 10.1254/jphs.fp0071924 18403899

[266] Gattaz WF Schummer B Behrens S Unit N Effects of zotepine, haloperidol and clozapine on MK-801-induced stereotypy and locomotion in rats. J Neural Transm Gen Sect (1994) 96:227–32. 10.1007/BF01294789 7826573

[267] Beraki S Kuzmin A Tai F Ögren SO Repeated low dose of phencyclidine administration impairs spatial learning in mice: blockade by clozapine but not by haloperidol. Eur Neuropsychopharm (2008) 18:486–97. 10.1016/j.euroneuro.2007.12.001 18242064

[268] Verma V Tan CH Ong WY Grigoryan GA Jones CA Stolzberg D The chakragati mouse shows deficits in prepulse inhibition of acoustic startle and latent inhibition. Neurosci Res (2008) 60:281–8. 10.1016/j.neures.2007.11.007 18164085

[269] Mutlu O Ulak G Celikyurt IK Akar FY Erden F Effects of olanzapine, sertindole and clozapine on learning and memory in the Morris water maze test in naive and MK-801-treated mice. Pharmacol Biochem Behav (2011) 98:398–404. 21334369 10.1016/j.pbb.2011.02.009

[270] Nakaya K Nakagawasai O Arai Y Onogi H Sato A Niijima F Pharmacological characterizations of memantine-induced disruption of prepulse inhibition of the acoustic startle response in mice: involvement of dopamine D2 and 5-HT2A receptors. Behav Brain Res (2011) 218:165–73. 10.1016/j.bbr.2010.11.053 21130810

[271] Nowakowska E Kus K Ratajczak P Cichocki M Wozniak A The influence of aripiprazole, olanzapine and enriched environment on depressant-like behavior, spatial memory dysfunction and hippocampal level of BDNF in prenatally stressed rats. Pharmacol Rep (2014) 66:404–11. 10.1016/j.pharep.2013.12.008 24905516

[272] Rajagopal L Massey BW Huang M Oyamada Y Meltzer HY The novel object recognition test in rodents in relation to cognitive impairment in schizophrenia. Curr Pharm Des (2014) 20:5104–14. 10.2174/1381612819666131216114240 24345269

[273] Celikyurt IK Akar FY Ulak G Mutlu O Erden F Effects of risperidone on learning and memory in naive and MK-801-treated rats. Pharmacology (2011) 87:187–94. 10.1159/000324523 21430408

[274] Gutierrez A Regan SL Hoover CS Williams MT Vorhees CV Effects of intrastriatal dopamine D1 or D2 antagonists on methamphetamine-induced egocentric and allocentric learning and memory deficits in Sprague–Dawley rats. Psychopharmacol (Berl) (2019) 236:2243–58. 10.1007/s00213-019-05221-3 PMC6626678 30919007

[275] Nordquist RE Risterucci C Moreau JL von Kienlin M Künnecke B Maco M Effects of aripiprazole/OPC-14597 on motor activity, pharmacological models of psychosis, and brain activity in rats. Neuropharmacology (2008) 54:405–16. 10.1016/j.neuropharm.2007.10.010 18054053

[276] Ishii D Matsuzawa D Kanahara N Matsuda S Sutoh C Ohtsuka H Effects of aripiprazole on MK-801-induced prepulse inhibition deficits and mitogen-activated protein kinase signal transduction pathway. Neurosci Lett (2010) 471:53–7. 10.1016/j.neulet.2010.01.010 20083164

[277] Vardigan JD Huszar SL McNaughton CH Hutson PH Uslaner JM MK-801 produces a deficit in sucrose preference that is reversed by clozapine, D-serine, and the metabotropic glutamate 5 receptor positive allosteric modulator CDPPB: relevance to negative symptoms associated with schizophrenia? Pharmacol Biochem Behav (2010) 95:223–9. 10.1016/j.pbb.2010.01.010 20122952

[278] Wedzony K Gołembiowska K Zazula M Differential effects of CGP 37849 and MK-801, competitive and noncompetitive NMDA antagonists, with respect to the modulation of sensorimotor gating and dopamine outflow in the prefrontal cortex of rats. Naunyn Schmiedebergs Arch Pharmacol (1994) 350:555–62. 10.1007/BF00173026 7870195

[279] Kesby JP Burne TH McGrath JJ Eyles DW Developmental vitamin D deficiency alters MK801-induced hyperlocomotion in the adult rat: an animal model of schizophrenia. Biol Psychiatry (2006) 60:591–6. 10.1016/j.biopsych.2006.02.033 16697353

[280] Sams-Dodd F Effects of dopamine agonists and antagonists on PCP-induced stereotyped behaviour and social isolation in the rat social interaction test. Psychopharmacol (Berl) (1998) 135:182–93. 10.1007/s002130050500 9497024

[281] Pietraszek M Ossowska K Chronic treatment with haloperidol diminishes the phencyclidine-induced sensorimotor gating deficit in rats. Naunyn Schmiedebergs Arch Pharmacol (1998) 357:466–71. 10.1007/pl00005194 9606034

[282] Rueter LE Ballard ME Gallagher KB Basso AM Curzon P Kohlhaas KL Chronic low dose risperidone and clozapine alleviate positive but not negative symptoms in the rat neonatal ventral hippocampal lesion model of schizophrenia. Psychopharmacol (Berl) (2004) 176:312–9. 10.1007/s00213-004-1897-4 15179541

[283] Shimazaki T Kaku A Chaki S D-serine and a glycine transporter-1 inhibitor enhance social memory in rats. Psychopharmacol (Berl) (2010) 209:263–70. 10.1007/s00213-010-1794-y 20198471

[284] Coutureau E Gosselin O Di Scala G Restoration of latent inhibition by olanzapine but not haloperidol in entorhinal cortex-lesioned rats. Psychopharmacol (Berl) (2000) 150:226–32. 10.1007/s002130000434 10907677

[285] Orsetti M Colella L Dellarole A Canonico PL Ghi P Modification of spatial recognition memory and object discrimination after chronic administration of haloperidol, amitriptyline, sodium valproate or olanzapine in normal and anhedonic rats. Int J Neuropsychopharmacol (2007) 10:345–57. 10.1017/S1461145706006705 16734936

[286] Chatterjee M Jaiswal M Palit G Comparative evaluation of forced swim test and tail suspension test as models of negative symptom of schizophrenia in rodents. ISRN Psychiatry (2012) 2012:595141. 23738205 10.5402/2012/595141 PMC3658575

[287] Noda Y Kamei H Mamiya T Furukawa H Nabeshima T Repeated phencyclidine treatment induces negative symptom-like behavior in forced swimming test in mice: imbalance of prefrontal serotonergic and dopaminergic functions. Neuropsychopharmacology (2000) 23:375–87. 10.1016/S0893-133X(00)00138-X 10989264

[288] Orsetti M Colella L Dellarole A Canonico PL Ferri S Ghi P Effects of chronic administration of olanzapine, amitriptyline, haloperidol or sodium valproate in naive and anhedonic rats. Int J Neuropsychopharmacol (2006) 9:427–36. 10.1017/S146114570500564X 15967060

[289] Torrisi SA Salomone S Geraci F Caraci F Bucolo C Drago F Buspirone counteracts MK-801-induced schizophrenia-like phenotypes through dopamine D3 receptor blockade. Front Pharmacol (2017) 8:1–13. 29046641 10.3389/fphar.2017.00710 PMC5632784

[290] Wang Y Yang X Song X Zhao L Wei J Wang J Co-treatment of buspirone with atypical antipsychotic drugs (AAPDs) improved neurocognitive function in chronic schizophrenia. Schizophr Res (2019) 209:135–40. 10.1016/j.schres.2019.05.006 31101513

[291] Kane J Honigfeld G Singer J Meltzer H Clozapine for the treatment-resistant schizophrenic. A Double-blind comparison with chlorpromazine. Arch Gen Psychiatry (1988) 45:789–96. 10.1001/archpsyc.1988.01800330013001 3046553

[292] Sommer IEC Slotema CW Daskalakis ZJ Derks EM Blom JD van der Gaag M The treatment of hallucinations in schizophrenia spectrum disorders. Schizophr Bull (2012) 38:704–14. 10.1093/schbul/sbs034 PMC3577047 22368234

[293] Svensson KA Heinz BA Schaus JM Beck JP Hao J Krushinski JH An allosteric potentiator of the dopamine D1 receptor increases locomotor activity in human D1 knock-in mice without causing stereotypy or tachyphylaxis. J Pharmacol Exp Ther (2017) 360:117–28. 10.1124/jpet.116.236372 PMC5193077 27811173

[294] Sison M Gerlai R Behavioral performance altering effects of MK-801 in zebrafish (*Danio rerio*). Behav Brain Res (2012) 220:331–7. 10.1016/j.bbr.2011.02.019 PMC3072452 21333690

[295] Zabegalov KN Khatsko SL Lakstygal AM Demin KA Cleal M Fontana BD Abnormal repetitive behaviours in zebrafish and their relevance to human brain disorders. Behav Brain Res (2019) 367:101–10. 10.1016/j.bbr.2019.03.044 30926483

[296] van den Buuse M Modeling the positive symptoms of schizophrenia in genetically modified mice: pharmacology and methodology aspects. Schizophr Bull (2010) 36:246–70. 10.1093/schbul/sbp132 PMC2833124 19900963

[297] Kumari V Peters ER Fannon D Premkumar P Aasen I Cooke MA Uncontrollable voices and their relationship to gating deficits in schizophrenia. Schizophr Res (2008) 101:185–94. 10.1016/j.schres.2007.12.481 PMC2845800 18262774

[298] Aleman A Lincoln TM Bruggeman R Melle I Arends J Arango C Treatment of negative symptoms: where do we stand, and where do we go? Schizophr Res (2017) 186:55–62. 27293137 10.1016/j.schres.2016.05.015

[299] Abekawa T Ito K Nakagawa S Nakato Y Koyama T Effects of aripiprazole and haloperidol on progression to schizophrenia-like behavioural abnormalities and apoptosis in rodents. Schizophr Res (2011) 125:77–87. 20833512 10.1016/j.schres.2010.08.011

[300] Wang L Zhang Y Wang C Zhang X Wang Z Liang X A natural product with high affinity to sigma and 5-HT7 receptors as novel therapeutic drug for negative and cognitive symptoms of schizophrenia. Neurochem Res (2019) 44:2536–45. 10.1007/s11064-019-02873-7 31529334

[301] Fulford D Campellone T Gard DE Social motivation in schizophrenia: how research on basic reward processes informs and limits our understanding. Clin Psychol Rev (2018) 63:12–24. 29870953 10.1016/j.cpr.2018.05.007

[302] Scheggi S Pelliccia T Gambarana C De Montis MG Aripiprazole relieves motivational anhedonia in rats. J Affect Disord (2017) 227:192–7. 10.1016/j.jad.2017.10.032 29100151

[303] Kanungo J Cuevas E Ali SF Paule MG Ketamine induces motor neuron toxicity and alters neurogenic and proneural gene expression in zebrafish. J Appl Toxicol (2013) 33:410–7. 10.1002/jat.1751 PMC5474752 22045596

